# High Light-Induced Nitric Oxide Production Induces Autophagy and Cell Death in *Chlamydomonas reinhardtii*

**DOI:** 10.3389/fpls.2020.00772

**Published:** 2020-06-10

**Authors:** Eva YuHua Kuo, Hsueh-Ling Chang, Shu-Tseng Lin, Tse-Min Lee

**Affiliations:** ^1^Department of Marine Biotechnology and Resources, National Sun Yat-sen University, Kaohsiung, Taiwan; ^2^Doctoral Degree Program in Marine Biotechnology, National Sun Yat-sen University, Kaohsiung, Taiwan

**Keywords:** autophagy, autophagy-related protein, cell death, *Chlamydomonas*, high light, nitric oxide

## Abstract

Autophagy plays a role in regulating important cellular functions in response to stress conditions. The role of nitric oxide (NO) in the regulation of autophagy in *Chlamydomonas reinhardtii* has been not studied. Illumination of *C. reinhardtii* cells under a high light (HL, 1,600 μmol m^–2^ s^–1^) condition induced a NO burst through NO synthase- and nitrate reductase-independent routes, and cell death. The abundance of CrATG8 protein, an autophagy marker of *C. reinhardtii*, increased after HL illumination along with a linear increase in the transcript abundance of autophagy-associated genes (CrVPS34, CrATG1, CrATG3, CrATG4, CrATG6, CrATG7, CrATG8, and CrATG12), which were suppressed in the presence of an NO scavenger, 2-(4-carboxyphenyl)-4,4,5,5-tetramethylimidazoline-1-oxyl-3-oxide (cPTIO). The cells were treated with NO donors, *S*-nitroso-*N*-acetyl-penicillamine, and *S*-nitrosoglutathione, under a normal light (50 μmol m^–2^ s^–1^) condition to elucidate the role of NO in autophagy activation and cell death. Treatment with 0.05 mM or 0.1 mM NO donors increased the abundance of ATG8 protein and CrATG transcripts, which were suppressed in the presence of cPTIO. Moreover, treatment with 0.05 mM NO donors did not affect cell viability, while 0.1 mM NO donors elicited a transient decrease in cell growth and death that recovered after 12 h. The transient effect could be prevented by the presence of cPTIO. However, treatment with 1 mM H_2_O_2_ and 0.1 mM NO donors enhanced autophagy induction and resulted in cell death after 24 h. The interaction of H_2_O_2_ and NO can be prevented by cPTIO treatment. This implies that NO is critical for the interaction of H_2_O_2_ and NO that induces cell death and autophagy. Furthermore, exposure to 0.1 mM NO donors under a non-lethal HL condition (750 μmol m^–2^ s^–1^) evoked autophagy and cell death. In conclusion, the present findings demonstrated that the NO-mediated autophagy pathway is activated in *C. reinhardtii* under lethal high intensity illumination and may interact with H_2_O_2_ for HL-induced cell death. The relationships between autophagy and cell death are discussed.

## Introduction

Nitric oxide (NO), a short-lived, gaseous molecule that can be either enzymatically or non-enzymatically synthesized in plants (Besson-Bard et al., 2008; Palavan-Unsal and Arisan, 2009), has been recognized as a novel biological messenger in the regulation of various biochemical and physiological activities and stress responses (Anbar, 1995; Neill et al., 2008; Hasanuzzaman et al., 2010; Hayat et al., 2010; Siddiqui et al., 2011; Bajguz, 2014). Recently, increasing importance has been attached to the role of NO in the regulation of high intensity light responses. However, whether NO plays a protective or a harmful role in the response of plants to excessive light energy depends on the plant species. Illumination of the leaves of tall fescue (*Festuca arundinacea* Schreb.) at an intensity of 500 μmol m^–2^ s^–1^ triggers NO production against oxidative stress by increasing the activity of antioxidant enzymes and the content of antioxidants (Xu et al., 2010). Foresia et al. (2010) has reported for a unicellular marine alga *Ostreococcus tauri* Gen et Sp-NOV that illumination at 400 μmol m^–2^ s^–1^ induces an NO burst, which is proposed to be a signal triggering a photoprotection mechanism against high light (HL)-induced oxidative damage. We have recently found a contrasting result in *Chlamydomonas reinhardtii* P.A. Dangeard that NO generated under very high intensity light (VHL; 3,000 μmol m^–2^ s^–1^) conditions is associated with VHL-induced cell death (Chang et al., 2013).

There is accumulating evidence that the generation of NO is crucial for the regulation of developmentally regulated and environmentally induced programmed cell death (PCD) in plants, either its promotion or its inhibition (Delledonne et al., 2001; Wang et al., 2013). NO delays the onset of cell death in gibberellin (GA)-induced PCD in barley aleurone layers (Beligni et al., 2002), while NO at high concentrations induces DNA fragmentation, membrane breakdown, and cell death (Pedroso et al., 2000; Yamasaki, 2000; Romero-Puertas et al., 2004). Moreover, NO is involved in the regulation of hypersensitive cell death (Clarke et al., 2000; de Pinto et al., 2002) and stress-induced cell death (Ahlfors et al., 2009; de Michele et al., 2009). NO also triggers cell death in algae; for example, the aldehyde-induced cell death in diatoms (Vardi et al., 2006), the heat-induced cell death of symbiotic alga *Symbiodinium microadriaticum* Freudenthal (Bouchard and Yamasaki, 2008), and the mastoparan (MP)-induced cell death of *C. reinhardtii* (Yordanova et al., 2010).

Reactive oxygen species (ROS) and oxidative stress modulate the autophagy process in plants (Pérez-Pérez et al., 2010, 2012b; Liu and Bassham, 2012; Bassham and Crespo, 2014). Stresses, including methyl viologen (MV)- or hydrogen peroxide (H_2_O_2_)-induced oxidative stress, nitrogen deficiency, carbon starvation by dark incubation, endoplasmic reticulum stress, and disordered chloroplast protein homeostasis due to a depletion of ClpP1 protease, are known to trigger autophagy in *C. reinhardtii* cells (Pérez-Pérez et al., 2010, 2012a,b, 2014; Ramundo et al., 2014). Moreover, a transfer of *C. reinhardtii* cells from dim light (5–10 μmol m^–2^ s^–1^) to high intensity light (1,200 μmol m^–2^ s^–1^) caused a transient increase of autophagy-related protein 8 (ATG8) abundance with a peak at 6 h, followed by a gradual decline to the control level when the high intensity illumination was prolonged to 24 h (Pérez-Pérez et al., 2012a). In comparison with wild type, the induction of autophagy by high intensity light illumination, MV, or H_2_O_2_, is more pronounced in *C. reinhardtii lts1-204* and *npq1 lor1* mutants, which exhibit a higher sensitivity to oxidative stress due to low carotenoid levels (Pérez-Pérez et al., 2012a).

Reactive nitrogen species (RNS) are also known to modulate autophagy. In animal system, NO activates autophagy in HeLa cells (Yang et al., 2008) and neurons (Barsoum et al., 2006) but suppresses autophagy in neurodegenerative diseases (Sarkar et al., 2011). In contrast, NO does not affect autophagy in cardiac myocytes (Rabkin and Klassen, 2007). This suggests that the differential regulation of autophagy by NO depends on the type of animal tissue. Apart from ROS and oxidative stress, the role of RNS in the control of autophagy has not previously been reported in *C. reinhardtii* cells, as far as we know. Therefore, the present study has examined whether NO modulates autophagy in *C. reinhardtii* cells under very high intensity illumination (HL, 1,600 μmol m^–2^ s^–1^), which can induce cell death. First, the time-course changes in NO production detected by 4-amino-5-methylamino-2′,7′-difluororescein (DAF-FM), the level of ATG8 detected using western blots, and the transcript abundance of autophagy-associated genes were determined. Furthermore, the role of NO was confirmed by experiments in the presence or absence of an NO scavenger, 2-(4-carboxyphenyl)-4,4,5,5-tetramethylimidazoline-1-oxyl-3-oxide (cPTIO). Then, the NO donors including *S*-nitrosoglutathione (GSNO) and *S*-nitroso-*N*-acetyl-penicillamine (SNAP) were treated under normal light (NL, 50 μmol m^–2^ s^–1^) illumination with or without cPTIO to confirm the role of NO in autophagy activation and cell death. Next, the NO donors were treated under non-lethal HL conditions (i.e., a moderate high light condition, ML, 750 μmol m^–2^ s^–1^) to examine whether NO can confer sensitivity of *C. reinhardtii* cells to the induction of autophagy and cell death under moderate high light illumination. In addition, the interaction of NO with H_2_O_2_ accumulated under HL illumination in the modulation of autophagy and cell death was investigated by the application of H_2_O_2_ together with SNAP or GSNO under NL conditions.

## Materials and Methods

### Algal Culture and Treatments

*Chlamydomonas reinhardtii* P.A. Dangeard strain CC125 (*mt*+) and CC124 (*mt*−) obtained from the Chlamydomonas Resource Center^1^ (University of Minnesota, St. Paul, MN, United States), were photoheterotrophically cultured in 50 mL of Tris-acetate phosphate (TAP) medium (Harris, 1989) containing a trace element solution in 125 mL flasks. The cultures were agitated on an orbital shaking incubator (model OS701, TKS company, Taipei, Taiwan) (150 rpm) under continuous illumination with a fluorescent white light at the NL intensity of 50 μmol m^–2^ s^–1^ at 28°C. After 18–24 h of incubation, algal cells that grew to the density of approximately 3 × 10^6^ cells mL^–1^ were centrifuged at 4,000 × *g* for 5 min at 28°C. The pellet was resuspended in fresh TAP medium as 3 × 10^6^ cells mL^–1^. Ten mL of the resuspended culture was transferred to a 100 mL beaker and incubated at 28°C under an NL condition for 1.5 h in an orbital shaker (model OS701, TKS Company, Taipei, Taiwan) at a speed of 150 rpm. Then, the algal cells were exposed to HL, 1,600 μmol m^–2^ s^–1^, or subjected to chemical treatments at 28°C. Each treatment had three independent biological replicates (*n* = 3) with each beaker as a biological replicate. The treatment has been repeated three times. Samples taken before (0 h) and after chemical treatment were imaged, then centrifuged at 4,000 × *g* for 5 min, and the pellet was fixed in liquid nitrogen and stored at −80°C until analysis.

To explore whether NO modulates the HL-induced autophagy and cell death, cPTIO was applied to scavenge NO at a final concentration of 400 μM. Because we have found that cPTIO lost its ability to scavenge NO after 2–3 h, 400 μM cPTIO was added again to the algal culture at 2.5 h. To mimic the effect of NO bursts under the HL condition, the NO donors, SNAP and GSNO (Sigma-Aldrich, St. Louis, MO, United States), were applied to the cells illuminated with 50 μmol m^–2^ s^–1^ intensity in the presence or absence of 400 μM cTPIO. In addition, to elucidate whether NO can promote the susceptibility of *C. reinhardtii* cells to high intensity illumination, SNAP and GSNO were applied separately at concentrations of 0.05 or 0.1 mM under a moderate high light condition (ML, 750 μmol m^–2^ s^–1^). In addition, the interaction of NO and H_2_O_2_ in the induction of autophagy and cell death was also investigated by treatment with 1 mM H_2_O_2_ with and without 0.1 mM NO donors under an NL condition for 3 h. In this experiment, the role of NO was confirmed by the treatment of cPTIO with an NO donor and H_2_O_2_.

In the attempts to elucidate the involvement of NO synthase (NOS) or nitrate reductase (NR) in the HL-induced NO production, the inhibitors of NOS and NR, *N*^ω^-nitro-L-arginine methyl ester (L-NAME), *N*^ω^-monomethyl-L-arginine (L-NMMA), and sodium tungstate, respectively, were added to the TAP medium 1.5 h before the HL treatment.

### Determination of Cell Density and Viability

For cell density estimation, 10 μL of algal culture was mixed with 30 μL of Lugol’s solution (Sigma-Aldrich, St. Louis, MO, United States) and the cell number was counted in duplicate using a light microscope (BX43, Olympus, Tokyo, Japan) and a hemocytometer (Improved Neubauer, Boeco, Germany). The cell density was calculated according to the manufacturer’s manual and expressed as units of 10^6^ mL^–1^.

After treatments, the viability of the algal cells was estimated by loading 2 μL of cell suspension on TAP agar plates, and incubating the plates for 72 h at 28°C under illumination at 50 μmol m^–2^ s^–1^ intensity. The colonies were imaged with a digital Nikon camera, and the final composite images were constructed using Adobe Photoshop (Adobe Systems, San Jose, CA, United States). Together with a cell density curve, the cell viability was assessed by growth ability (the color and size of the colony) and was used to evaluate the effects of HL illumination or chemical challenges.

### Detection of Dead Cells Using Sytox Green Fluorescence

Cell death was assessed using the SYTOX-Green fluorescent probe (Molecular Probes Inc., Eugene, OR, United States). The SYTOX-Green stock solution of 5 mM in 100% DMSO was added to 1 mL of algal culture at a final concentration of 5 μM, and the mixture was incubated for 5 min at room temperature in the dark. The fluorescence level was detected by a fluorescence spectrophotometer at 525 nm (excitation: 488 nm) (Sato et al., 2004). Based on the blank (TAP medium without algal cells), the relative SYTOX-Green fluorescence level was estimated and expressed as relative fluorescence⋅(10^6^ cells)^–1^.

Then, the cells were observed with a fluorescence microscope (Eclipse Ni, Nikon, Tokyo, Japan) with excitation at 488 nm using FITC (excitation wavelength: 465–495 nm, emission wavelength: 515–555 nm) and B-2A (excitation wavelength: 450–490 nm, emission wavelength: >520 nm) filters (Nikon, Tokyo, Japan). Fluorescent images were acquired using a CCD camera (Nikon’s Digital Sight DSU3, Tokyo, Japan) and imported into Adobe Photoshop.

### Detection of NO Production

An NO-sensitive fluorescent dye, DAF-FM diacetate (Kojima et al., 1998), purchased from Invitrogen Life Technologies (Carlsbad, CA, United States) was employed to measure NO production according to the procedure described in our previous study (Chang et al., 2013). DAF-FM diacetate was preloaded into cell cultures prior to HL and chemical treatments. Algal cells (1 mL) were preincubated in TAP medium containing 5 μM DAF-FM diacetate for 60 min at 25°C under NL to allow the penetration of DAF-FM acetate into the cells (Chang et al., 2013). Then, the algal cells were washed twice with fresh TAP medium and exposed to HL or treated with a chemical. The fluorescence in the cells was determined with a fluorescence spectrophotometer (excitation/emission: 492 nm/525 nm; F-2500, Hitachi, Tokyo, Japan) and observed with a fluorescence microscope (Eclipse Ni, Nikon, Tokyo, Japan) using a FITC filter (Nikon, Tokyo, Japan). Based on the fluorescence of the blank (TAP medium without algal cells), the relative DAF-FM fluorescence level was estimated and expressed as relative fluorescence⋅(10^6^ cells)^–1^.

### Protein Extraction and Western Blots

Soluble protein was extracted according to Pérez-Pérez et al. (2012a). After treatment for 3 h under NL or HL conditions, algal cells (*n* = 3, three independent biological replicates) were collected by centrifugation (4,000 × *g*, 5 min), washed once with lysis buffer (50 mM Tris-HCl, pH 7.5) and resuspended in 50 μL lysis buffer. Cells were lysed by two cycles of slow freezing to −80°C, followed by fast thawing to room temperature. The soluble cell extract was then separated from the insoluble fraction by centrifugation (15,000 × *g*, 15 min) at 4°C and immediately used for western-blot analysis and protein quantitation. Protein concentrations were quantified by the Coomassie Blue dye binding method (Bradford, 1976) using the concentrated dye purchased from BioRad (500-0006, Hercules, CA, United States). Samples were heated to 100°C for 5 min and then used for western blot analysis. For each sample, 30 μg of protein was loaded into each lane, resolved by 15% SDS-PAGE, and transferred to a polyvinylidene fluoride membrane for antibody binding. After being blocked with 5% BSA (Sigma, St. Louis, MO, United States), the membranes were incubated with a rabbit polyclonal anti-ATG8 antibody (ab77003; Abcam, Cambridge, United Kingdom) and a mouse monoclonal antibody against α-tubulin (ab11304; Abcam, Cambridge, United Kingdom) for 90 min at room temperature. After being washed, the membranes were incubated for 90 min with horseradish peroxidase-conjugated secondary antibodies (MD20878; KPL, Gaithersburg, MD, United States), and then the immunoblots were visualized using the ECL Plus chemiluminescence system (Thermo Scientific Inc., Chicago, IL, United States). A LAS-3000 Mini Lumino-image Analyzer (LAS-3000, Fujifilm, Tokyo, Japan) was used for imaging the chemiluminescent western blots. The relative abundance of CrATG8 protein was estimated on the basis of α-tubulin intensity.

### RNA Isolation, cDNA Synthesis, and mRNA Quantification by Real-Time Quantitative PCR

For RNA isolation, the algal cells in 5 mL of culture sampled from each of the independent biological replicates (*n* = 3) were harvested by centrifugation as described above. Total RNA was extracted with TriPure Isolation Reagent (Roche Applied Science, Mannheim, Germany) according to the manufacturer’s instructions. The integrity of the RNA was checked by visual inspection of the two ribosomal RNAs, 18S and 28S, on an ethidium bromide stained 1% agarose (MDBio Inc., Taipei, Taiwan) gel. The RNA sample concentration was adjusted to 2.95 μg total RNA⋅μL^–1^. After treatment with DNase (TURBO DNA-*free*TM Kit, Ambion Inc., The RNA Company, United States) to remove residual DNA, 1.5 μg of total RNA was used for the preparation of cDNA following the protocol previously described (Chang et al., 2013). The cDNA was amplified from the poly-(A+) end using oligo (dT) 12–18 from a VersoTM cDNA Kit (Thermo Fisher Scientific Inc., Waltham, MA, United States). The volume was adjusted to provide a concentration of 30 ng mL^–1^ of the original RNA for each sample. The primers for the CrTOR, CrVSP34, and CrATG genes (Supplementary Table S1) were designed according to the Phytozome genome using LightCycler Probe Design2 (Roche Applied Science, Mannheim, Germany). The real-time quantitative PCR was performed using the LightCycler 480 system (Roche Applied Science, Mannheim, Germany). A master mix for PCR was prepared with a LightCycler^®^ 480 SYBR Green I Master Kit (Roche Applied Science, Mannheim, Germany). To optimize the primer concentration, real-time PCR analyses using different primer concentrations and a constant template cDNA concentration of 30 or 50 ng were performed. To optimize the cDNA template concentration, each pair of primers was tested across a log dilution series of a positive control DNA sample. After optimization of the real-time PCR conditions, a primer concentration of 3 μM and a cDNA template concentration of 30 ng μL^–1^ were used for the detection of CrATG transcript abundance, and a primer concentration of 6 μM and a cDNA template concentration of 30 ng μL^–1^ were used for the detection of the CrVSP34 transcript abundance. Each reaction was performed in a total volume of 10 μL, which contained LightCycler^®^ 480 SYBR Green I Master Mix, the selected concentration of each primer, and cDNA corresponding to 30 or 50 ng μL^–1^ RNA in the reverse transcriptase reaction. The amplification program was started by first denaturation at 95°C for 5 min, followed by 50 amplification cycles of annealing at 60°C for 10 s, and elongation at 72°C for 5 s for real time fluorescence measurements. The dissociation curves were performed after the PCR, and the fluorescence was analyzed using the LightCycler 480 system. Software with auto CT (cycle threshold) was used to determine the threshold of each gene, and the 2^–Δ^ ^Δ^ ^CT^ method was used to calculate CT values, in which the relative change in transcript abundance was normalized to reference genes, ubiquitin-conjugating enzyme E2 isoform (*CrUBC*, NCBI: AY062935) and elongation factor 1 alpha (*CrEF-1α*, NCBI: XM_001696516.1) (see Supplementary Table S1 for the primers). The fold increase was calculated relative to the RNA sample from the control without light or chemical treatment at 0 h. Because the results based on the *CrEF-1α* internal control were similar to those based on *CrUBCX*, the relative changes in the transcript abundance on the basis of *CrUBCX* are shown in the present manuscript. All of the results presented are the averages of three independent biological replicates.

### Statistics

Three independent biological replicates (*n* = 3) were used for each experiment and all of the experiments were repeated at least three times. Because they showed a similar trend, only the results from one of them are shown in this paper. Statistical analyses were performed using SPSS (SPSS 15.0 for Windows Evaluation Version, Chicago, IL, United States). Significant differences between sample means were analyzed using Student’s *t*-test or Duncan’s new multiple range test and a subsequent significant analysis of variance for the controls and treatments at *P* < 0.05.

## Results

### HL Illumination Induces NO Burst and Cell Death

**FIGURE 1:**
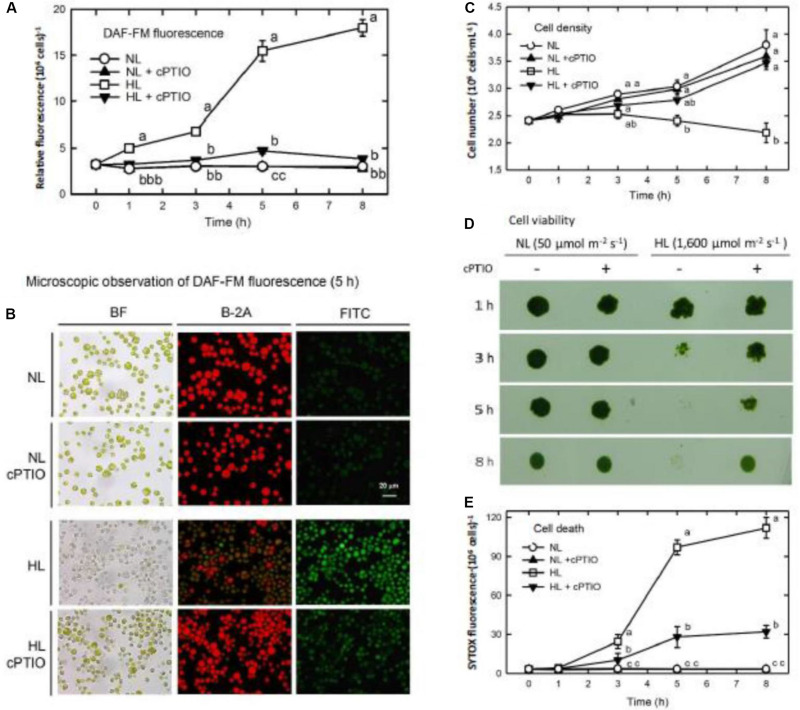
DAF-FM acetate detection of NO production, cell growth, and death in *Chlamydomonas reinhardtii* cells under NL (50 μmol m^–2^ s^–1^) and HL (1,600 μmol m^–2^ s^–1^). **(A)** Quantitation of NO production. **(B)** Microscopic observation of DAF-FM fluorescence after 5 h of treatment. **(C)** Cell density. **(D)** Cell viability. **(E)** Cell death (SYTOX Green fluorescence). The data in **(A)**, **(C)**, and **(E)** are expressed as the mean ± SD (*n* = 3) from three independent biological replicates, and different letters indicate the statistical significance set at *P* < 0.05 according to ANOVA. In **(B)**, BF represents bright field, B-2A represents the autofluorescence of cells, and FITC represents DAF-FM fluorescence.

An NO-sensitive fluorescent dye, DAF-FM diacetate, was loaded into the TAP medium before CC125, the *nit1 nit2* mt− genotype that cannot use nitrate and nitrite, was transferred to a HL condition. The production of NO increased linearly during 1–3 h after HL illumination, followed by a significant increase at 5 h and a small increment during 5–8 h (Figure 1A). Most of the algal cells observed under the microscope bleached 5 h after HL illumination and concomitantly emitted intense DAF-FM fluorescence (NO) (Figure 1B). Whether the DAF-FM fluorescence was attributable to NO was confirmed by the treatment with an NO scavenger, cPTIO (400 μM). cPTIO was added twice, at 0 and 2.5 h, to remove the NO generated during HL illumination. Although cPTIO can cause an increase in the DAF-FM fluorescence under certain conditions due to oxidization of NO to the NO_2_ radical with the subsequent formation of N_2_O_3_ (NO_2_ + NO → N_2_O_3_), which can react with the DAF-FM dye to form the fluorescent DAF-2T (Mur et al., 2011; D’Alessandro et al., 2013), our current results showed that the presence of cPTIO effectively reduced the DAF-FM fluorescence in the HL-treated cells (Figures 1A,B). This provides evidence supporting the hypothesis that DAF-FM fluorescence emittance under the HL condition is a result of NO.

The level of DAF-FM fluorescence determined in this study represented the cumulative NO production because the DAF-FM dye was loaded prior to the treatment. Thus, the NO production rate can be estimated from the difference in relative fluorescent units (RFU) between the two time points. As calculated from the data shown in Figure 1A, the NO production rate of NL-grown cells was 0 RFU h^–1^ over the incubation time, whereas that of the HL-treated cells was 0, 1.43, 0.98, 2.85, and 0.52 RFU h^–1^ from 0–0.5, 0.5–1, 1–3, 3–5, and 5–8 h, respectively. It is obvious that a transfer of *C. reinhardtii* cells to a HL condition triggers transient NO production with a profound burst when the time of exposure extends from 0.5 to 5 h with the maximum released during 3–5 h, followed by a decrease.

The cell growth expressed as cell density was inhibited upon exposure to HL illumination (Figure 1C), in which the cell viability decreased after 3 h and then the cells completely died after 5 h (Figure 1D). Similarly, using SYTOX-Green dye staining, the extent of dead cells increased 3 h after HL illumination and reached the maximum after 5 h (Figure 1E). The presence of cPTIO can prevent the HL-induced cell bleaching [see bright field (BF) in Figure 1B], growth inhibition (Figures 1C,D), and cell death (Figure 1E). The effect of cPTIO in the mitigation of HL-induced cell death demonstrates that the large quantity of NO released during prolonged HL illumination is associated with cell death and, in turn, results in a decline in cell growth.

We found that the illumination with moderate light intensity of 750 μmol m^–2^ s^–1^ did not induce NO generation (DAF-FM fluorescence) during a 24 h period (Supplementary Figure S1A) and the algal cells can acclimate to this moderate light intensity illumination, reflected by normal growth ability the same as algal cells grown under the NL condition (Supplementary Figure S1B). The extent of cell death evaluated by SYTOX-Green staining was slightly under 750 μmol m^–2^ s^–1^ illumination compared to significant cell death in the 1,600 μmol m^–2^ s^–1^ condition (Supplementary Figure S1C and Figure 1E). These correlative results reveal that the burst of NO is dependent on the light intensity and the NO production appears to have a negative relationship with cell molarity.

**FIGURE 2:**
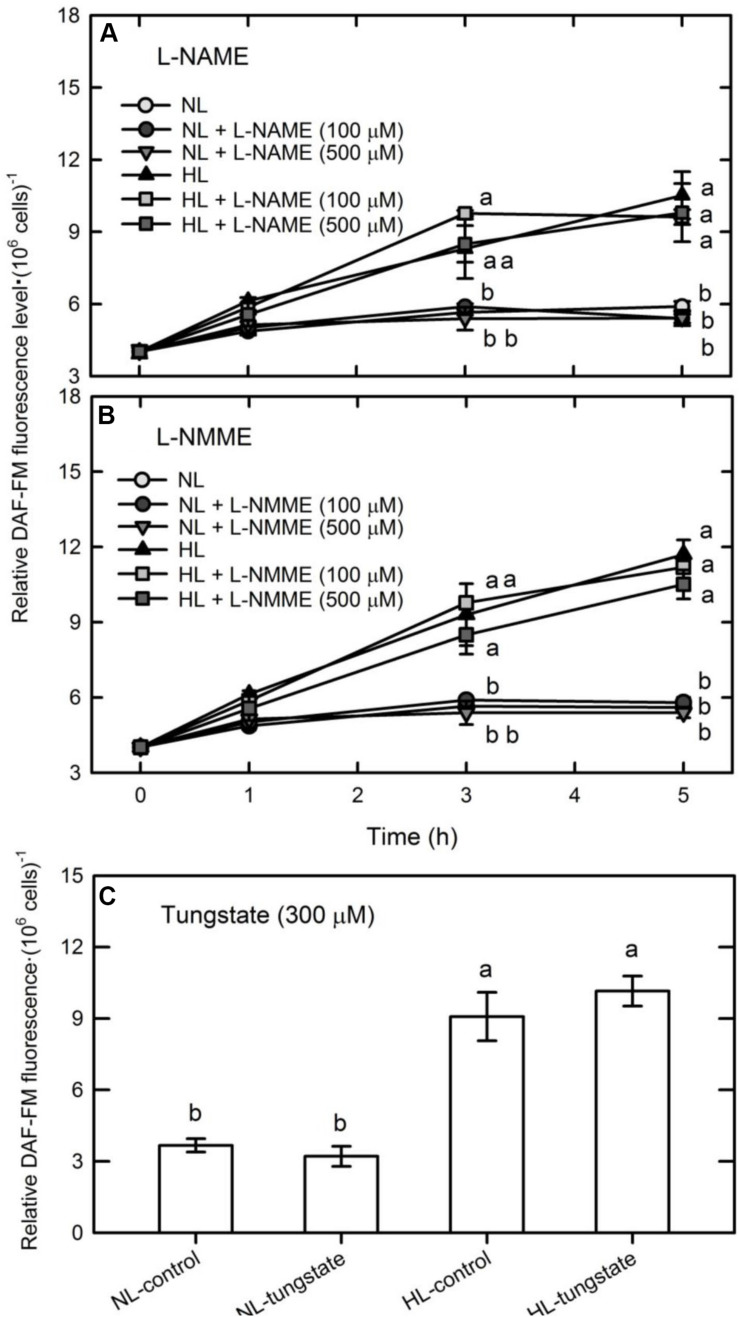
The effect of the NOS inhibitor, L-NAME **(A)** or L-NMME **(B)**, and the NR inhibitor, tungstate (300 μM) **(C)**, on NO production in *Chlamydomonas reinhardtii* cells under NL (50 μmol m^–2^ s^–1^) or HL (1,600 μmol m^–2^ s^–1^) illumination. The data are expressed as the mean ± SD (*n* = 3) from three independent biological replicates and different letters indicate the statistical significance set at *P* < 0.05 according to ANOVA.

Nitric oxide can be synthesized in plants by an enzymatic process involving NOS or NR, or by non-enzymatic reactions (Besson-Bard et al., 2008; Palavan-Unsal and Arisan, 2009). Whether NO was synthesized via NOS- or NR-dependent routes in *C. reinhardtii* cells under HL illumination was studied by treating them with the NOS inhibitors, L-NAME (100 or 500 μM) and L-NMMA (100 or 300 μM), and the NR inhibitor, tungstate (300 μM). These inhibitors did not affect the DAF-FM fluorescence of NL- or HL-treated cells (Figure 2). We have also found that the *C. reinhardtii* strain CC124 (*nit1 nit2 mt*+) that lacks NR and cannot grow in a medium containing nitrate or nitrite exhibited a similar NO production pattern under HL illumination (Supplementary Figure S2). Because *C. reinhardtii* strains CC125 and CC124 are mutants lacking NR and cannot use nitrate and nitrite, this implies that the HL-induced NO burst is independent of NR. Therefore, our present findings indicate that neither NOS nor NR is involved in the synthesis of NO in the HL-treated *C. reinhardtii* cells. This agrees with the results of our previous study that NO generated in *C. reinhardtii* cells exposed to an extremely high-intensity illumination (3,000 μmol m^–2^ s^–1^) is derived from sources other than the NOS- or NR-mediated pathways (Chang et al., 2013).

### NO Involvement of Autophagy Induction Under HL Illumination

**FIGURE 3:**
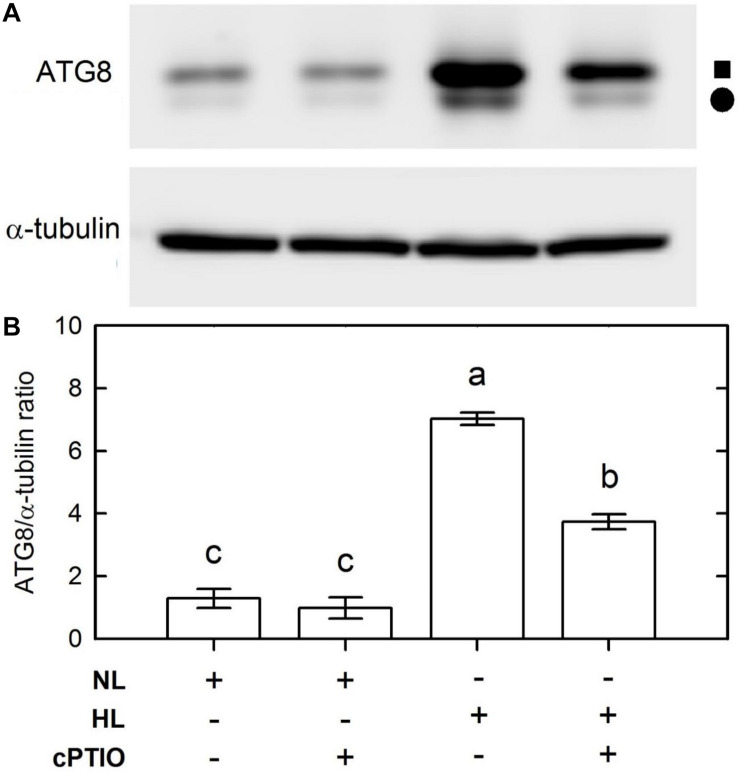
Immunodetection of the CrATG8 protein of *Chlamydomonas reinhardtii* cells 3 h after NL (50 μmol m^–2^ s^–1^) and HL (1,600 μmol m^–2^ s^–1^) illumination in the presence or the absence of 400 M cPTIO. **(A)** Immunodetection using an antibody directed against recombinant Arabidopsis ATG8 (upper part) and α-tubulin (lower part). Triplicate samples were analyzed, and one of them is shown. **(B)** Relative change of CrATG8 protein expression estimated on the basis of the α-tubulin intensity. A solid square (■) represents ATG8 and a solid circle (∙) represents ATG8-PE. The data in **(B)** are expressed as the mean ± SD (*n* = 3) from three independent biological replicates, and different letters indicate the statistical significance set at *P* < 0.05 according to ANOVA.

Because the CrATG8 protein was recognized as an autophagy marker of *C. reinhardtii* (Pérez-Pérez and Crespo, 2010), a commercial ATG8 antibody raised using recombinant *Arabidopsis thaliana* APG8A (Abcam, Cambridge, United Kingdom) was used in western blot assays to estimate the abundance of the CrATG8 protein. On a 15% gel, two protein bands with apparent molecular masses of 15 and 14.6 kD were detected in the soluble extract [the solid square symbol represents ATG and the solid circle symbol represents ATG-PE (phosphatidylethanolamine)] (Figure 3). Based on the 0 h control, the abundance of both CrATG8 and CrATG8-PE detected on western blot showed an apparent increase by 3 h of HL treatment (Figure 3A). Based on the normalization by α-tubulin intensity, we found that the relative abundances of CrATG8 and CrATG8-PE remained unchanged for the cells grown under the NL condition over a 0–3 h period while it increased seven-fold 3 h after HL illumination (Figure 3B). The HL-induced increase in the level of CrATG8 protein can be suppressed by the presence of cPTIO (Figure 3). ATG8 plays an essential role in the development of autophagosome formation, target recognition, and vacuole tethering (Mizushima et al., 2011) and is anchored to the autophagosome membrane through covalent binding to the membrane lipid PE to execute its function in autophagosome formation. The present result indicates that the expression of functional ATG8 is activated by accumulated NO under an HL condition.

**FIGURE 4:**
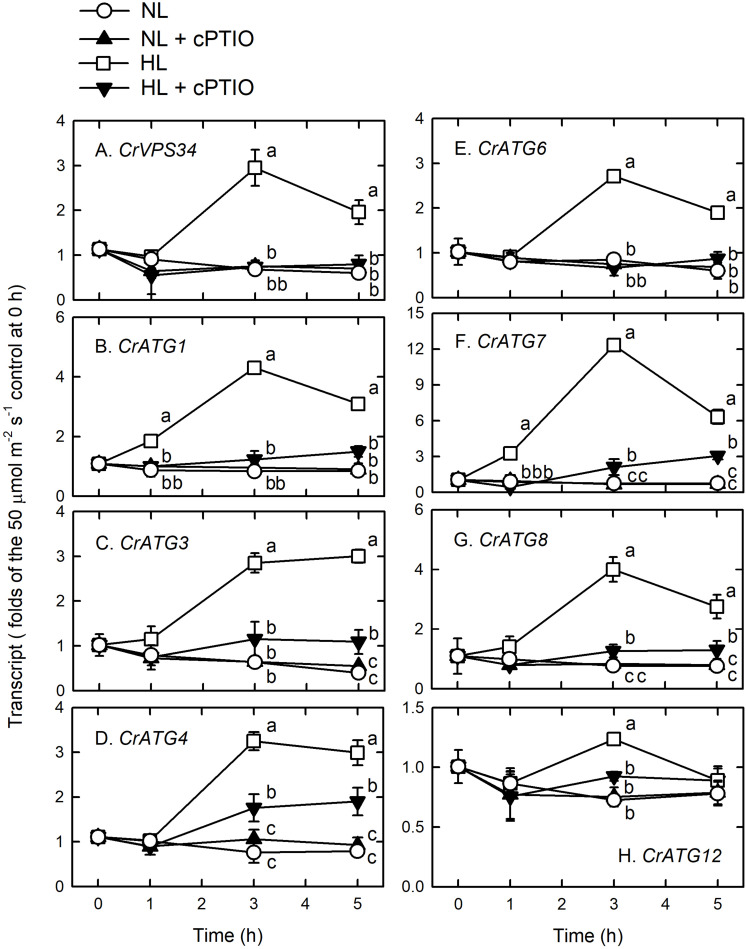
CrVPS34 and CrATG transcripts abundance in *Chlamydomonas reinhardtii* cells under NL (50 μmol m^–2^ s^–1^) and HL (1,600 μmol m^–2^ s^–1^) illumination in the presence or the absence of 400 μM cPTIO. **(A)** CrVPS34. **(B)** CrATG1. **(C)** CrATG3. **(D)** CrATG4. **(E)** CrATG6. **(F)** CrATG7. **(G)** CrATG8. **(H)** CrATG12. The data are expressed as the mean ± SD (*n* = 3) from three independent biological replicates. Different letters indicate the statistical significance set at *P* < 0.05 according to ANOVA. Open circle, NL; solid triangle, NL + cPTIO; open square, HL; inverted triangle, HL + cPTIO.

High light illumination immediately induced a significant increase in the transcript abundance of CrVPS34 (Figure 4A), CrATG1 (Figure 4B), CrATG3 (Figure 4C), CrATG4 (Figure 4D), CrATG6 (Figure 4E), CrATG7 (Figure 4F), and CrATG8 (Figure 4G), with a peak at 3 h. For CrATG12, although its transcript abundance was statistically increased by HL illumination, it only showed a 1.3-fold increase compared to the NL control (Figure 4H). The presence of cPTIO, which scavenged NO generated under the HL condition (Figure 1A), could suppress the HL-induced increase of CrVPS34 and CrATG transcripts abundance (Figure 4). Accordingly, the HL-induced CrVPS34 and CrATG gene expression is mediated by NO generated under HL illumination.

### Treatment With NO Donors Induces Autophagy Under NL Conditions

The role of NO in the modulation of autophagy was further confirmed by the treatment with NO donors, GSNO (0.05 or 0.1 mM) and SNAP (0.05 or 0.1 mM), under NL conditions. The time-course survey of the level of NO released showed that NO was rapidly emitted within 0.5 h after SNAP (Supplementary Figure S3A) or GSNO (Supplementary Figure S3B) treatment and reached a plateau after 1 h. Compared to the treatment with 0.05 mM, the release of NO was approximately two-fold higher for the treatment with 0.1 mM NO donors. The NO released from GSNO or SNAP at either the 0.05 or 0.1 mM concentration can be scavenged in the presence of 400 μM cPTIO (Supplementary Figure S3). The concentrations and the time-course changes of NO released from 0.1 mM SNAP to 0.1 mM GSNO were similar to those occurring under HL conditions. Therefore, to identify the effect of NO on autophagy and cell death without the interference of HL illumination, in the NL condition, NO donor, SNAP, or GSNO was applied at concentrations of either 0.05 or 0.1 mM.

**FIGURE 5:**
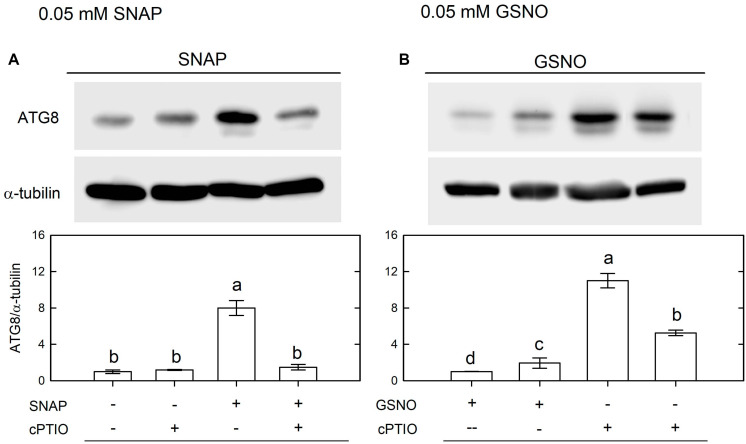
Immunodetection of the CrATG8 protein of *Chlamydomonas reinhardtii* cells after 0.05 mM SNAP **(A)** or 0.05 mM GSNO **(B)** treatment for 3 h in the presence or absence of 400 μM cPTIO under an NL (50 μmol m^–2^ s^–1^) condition. Triplicate samples were analyzed, and one of them is shown. The quantitation data are expressed as the mean ± SD (*n* = 3) from three independent biological replicates and different letters indicate the statistical significance set at *P* < 0.05 according to ANOVA.

**FIGURE 6:**
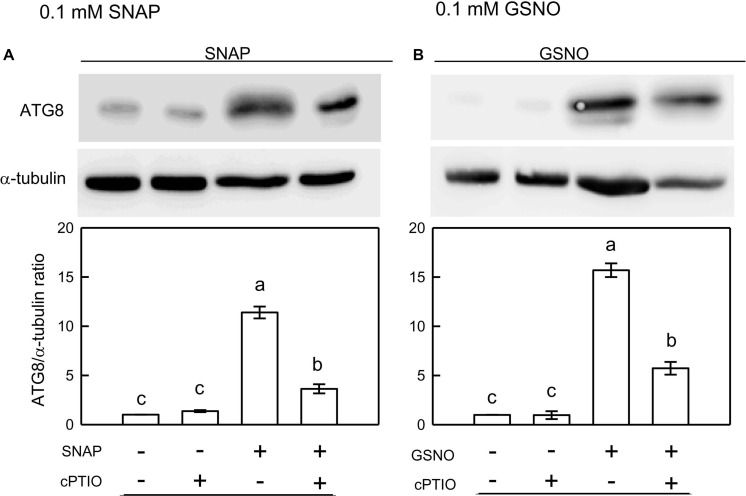
Immunodetection of the CrATG8 protein of *Chlamydomonas reinhardtii* cells after 0.1 mM SNAP **(A)** or 0.1 mM GSNO **(B)** treatment for 3 h in the presence or absence of 400 μM cPTIO under an NL (50 μmol m^–2^ s^–1^) condition. Triplicate samples were analyzed, and one of them is shown. The quantitation data are expressed as the mean ± SD (*n* = 3) from three independent biological replicates and different letters indicate the statistical significance set at *P* < 0.05 according to ANOVA.

**FIGURE 7:**
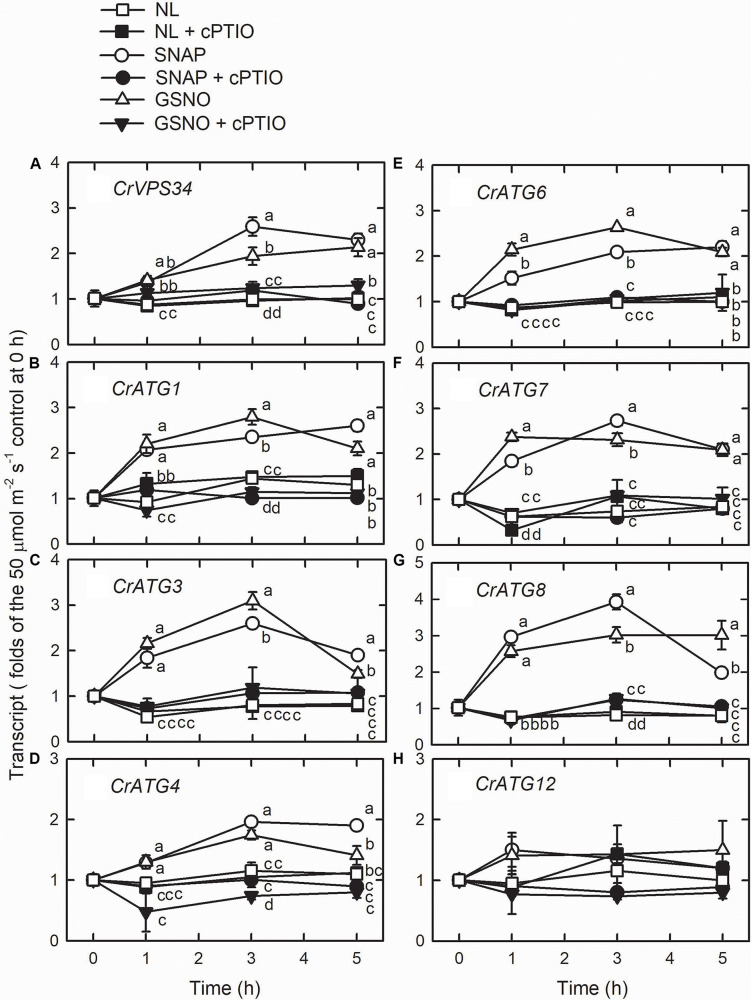
The time-course changes in CrVPS34 and CrATG transcripts abundance in *Chlamydomonas reinhardtii* cells in response to 0.05 mM SNAP or 0.05 mM GSNO under an NL (50 μmol m^–2^ s^–1^) condition. **(A)** CrVPS34. **(B)** CrATG1. **(C)** CrATG3. **(D)** CrATG4. **(E)** CrATG6. **(F)** CrATG7. **(G)** CrATG8. **(H)** CrATG12. The data are expressed as the mean ± SD (*n* = 3) from three independent biological replicates and different letters indicate the statistical significance set at *P* < 0.05 according to ANOVA.

**FIGURE 8:**
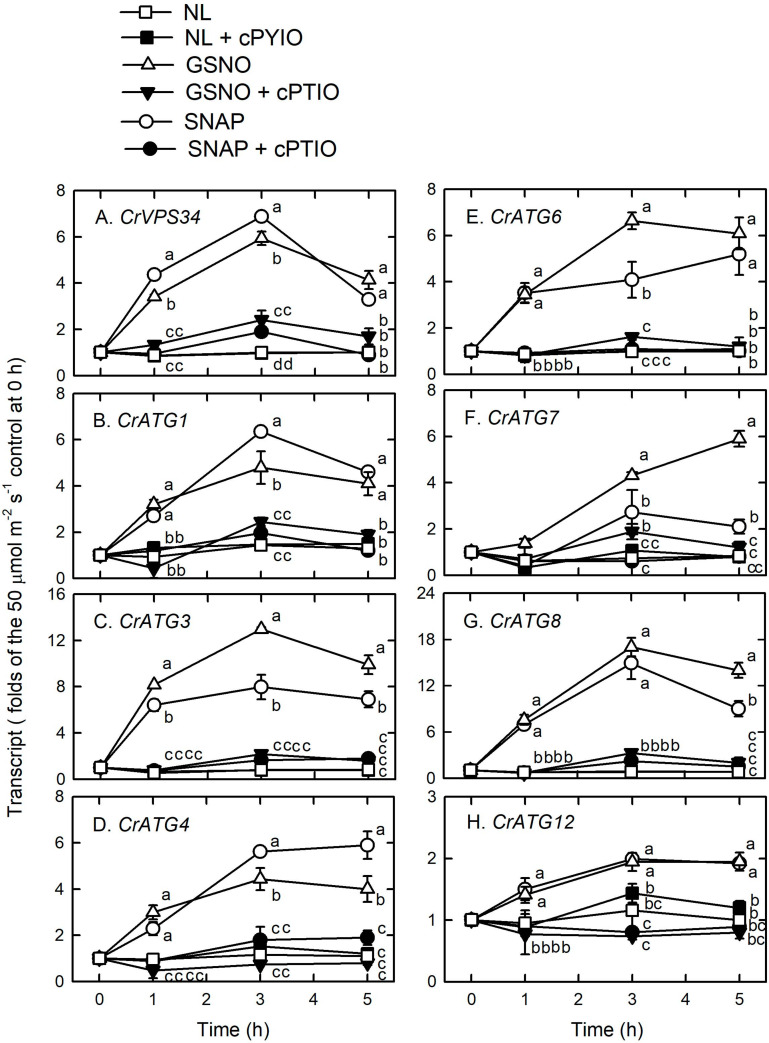
The time-course changes in CrVPS34 and CrATG transcripts abundance in *Chlamydomonas reinhardtii* cells in response to 0.1 mM SNAP or 0.1 mM GSNO under an NL (50 μmol m^–2^ s^–1^) condition. **(A)** CrVPS34. **(B)** CrATG1. **(C)** CrATG3. **(D)** CrATG4. **(E)** CrATG6. **(F)** CrATG7. **(G)** CrATG8. **(H)** CrATG12. The data are expressed as the mean ± SD (*n* = 3) from three independent biological replicates and different letters indicate the statistical significance set at *P* < 0.05 according to ANOVA.

Using western blot assays, the abundance of CrATG8 protein was markedly increased by the treatment with 0.05 mM SNAP (Figure 5A) or 0.05 mM GSNO (Figure 5B) under the NL condition for 3 h. When treated with a higher concentration (0.1 mM), the CrATG8 protein exhibited a higher increment than with 0.05 mM treatment (Figure 6). We also found that the expression of genes related to autophagy were induced by exposure to 0.05 mM (Figure 7) and 0.1 mM (Figure 8) NO donors. In response to 0.05 mM NO donors, the transcript abundances of CrVPS34 (Figure 7A), CrATG1 (Figure 7B), CrATG3 (Figure 7C), CrATG4 (Figure 7C), CrATG6 (Figure 7E), CrATG7 (Figure 7F), and CrATG8 (Figure 7G) increased approximately 3 h after treatment, but these treatments did not affect the CrATG12 transcript abundance (Figure 7H). When the concentration of NO donors was increased to 0.1 mM, the transcript abundances of CrVPS34 and all CrATG genes showed a higher induction compared to 0.05 mM treatment (Figure 8). The transcript abundance of CrATG12 can be slightly increased by 0.1 mM NO donors (Figure 8H) but not by a lower NO donor concentration (Figure 7H). The presence of cPTIO can suppress the SNAP- or GSNO-induced increase in CrATG8 protein abundance (Figures 5, 6) and the transcripts abundance of the CrVPS34 and CrATG genes (Figures 7, 8). The present findings demonstrated that NO released from NO donors can elicit autophagy in *C. reinhardtii* cells grown under NL conditions.

### Treatment With NO Donors at Higher Concentrations Under the NL Condition Causes Cell Death

**FIGURE 9:**
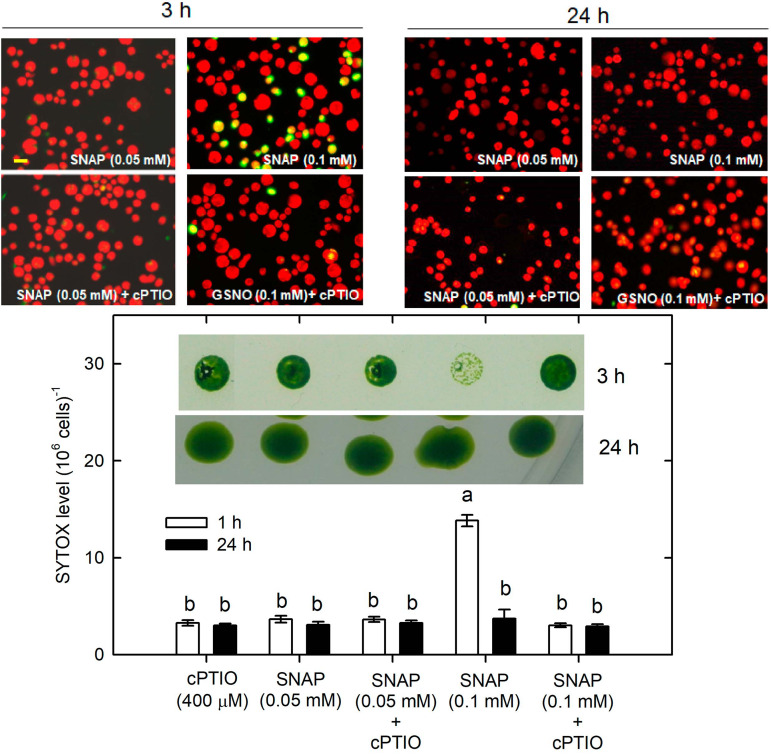
The effect of 0.05 **(A)** or 0.1 **(B)** mM SNAP treatment on cell viability and cell death detected by SYTOX Green fluorescence dye in *C. reinhardtii* cells under an NL (50 μmol m^–2^ s^–1^) condition in the presence or the absence of 400 μM cPTIO. The results for 3 and 24 h treatment are shown. The cell viability shown is after the treatment with 0.1 mM NO donors. The data are expressed as the mean ± SD (*n* = 3) from three independent biological replicates and the vertical bar on the symbols represents SD. Different letters indicate the statistical significance set at *P* < 0.05 according to ANOVA.

**FIGURE 10:**
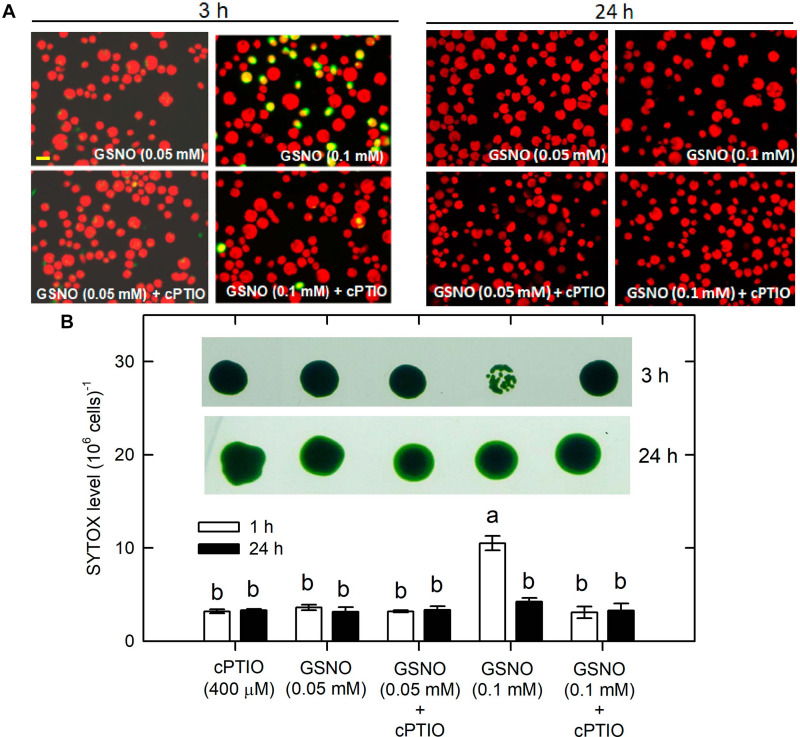
The effect of 0.05 **(A)** or 0.1 **(B)** mM GSNO treatment on cell viability and cell death detected by SYTOX Green fluorescence dye in *C. reinhardtii* cells under an NL (50 μmol m^–2^ s^–1^) condition in the presence or absence of 400 μM cPTIO. The results for 3 and 24 h treatment are shown. The cell viability shown is after the treatment with 0.1 mM NO donors. The data are expressed as the mean ± SD (*n* = 3) from three independent biological replicates and the vertical bar on the symbols represents SD. Different letters indicate the statistical significance set at *P* < 0.05 according to ANOVA.

Whether NO can lead to cell death under the NL condition was determined. The treatment with 0.05 mM SNAP or 0.05 mM GSNO did not affect cell growth (Supplementary Figure S7) or cell viability (Figure 9 for SNAP and Figure 10 for GSNO). However, when the NO donor concentration was increased to 0.1 mM, the cell growth was temporarily inhibited during the 3–5 h after treatment but then it could be recovered to the control level after a prolonged growth period (12–24 h) (Supplementary Figure S7). Furthermore, we also observed that both 0.1 mM SNAP (Figure 9) and 0.1 mM GSNO (Figure 10) treatment for 3 h triggered a minor manifestation of cell death symptoms as reflected by the appearance of SYTOX Green fluorescence in some algal cells, and then the fluorescence was no longer observed after 24 h. In addition, the temporary decrease of cell viability and the induction of cell death by 0.1 mM NO donors at 3 h could be prevented by the presence of cPTIO (Figures 9, 10, the viability in the Figures was for 0.1 mM SNAP or GSNO treatment). The cell viability at 0.05 mM was not affected (data not shown). This demonstrates that the transient viability decline and cell death were caused by higher NO release from 0.1 mM SNAP or 0.1 mM GSNO, but it did not occur under a lower NO donor concentration of 0.05 mM.

### Treatment With NO Donors Under a Moderate HL Condition Causes Cell Death and Stimulates Autophagy Activation

Subsequently, in the attempt to elucidate whether the excessive burst of NO confers the susceptibility of *C. reinhardtii* cells to non-lethal HL illumination in terms of autophagy induction and cell death, the NO donors were applied under a ML condition (750 μmol m^–2^ s^–1^). An exposure to ML illumination did not induce NO production (Supplementary Figure S1A), but it triggered a linear increase in H_2_O_2_ production as the treatment time advanced (Supplementary Figure S6). However, the level of ML-induced H_2_O_2_ production was lower than that under the 1,600 μmol m^–2^ s^–1^ condition (Supplementary Figure S6). Furthermore, we found that although H_2_O_2_ was produced under ML illumination, the algal cells exhibited a similar growth ability to those grown under an NL condition (Supplementary Figure S1B). This indicates that *Chlamydomonas* cells can tolerate accumulated H_2_O_2_ and acclimate to moderate light stress.

**FIGURE 11:**
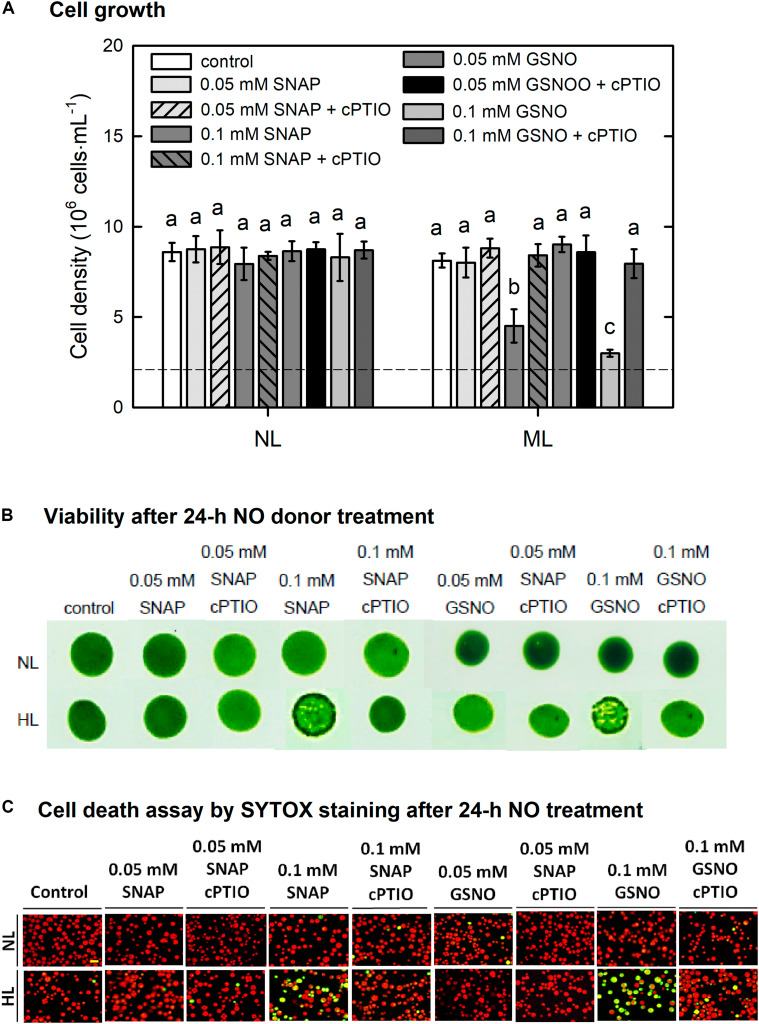
The effect of 0.05 or 0.1 mM SNAP and GSNO treatment on cell growth **(A)**, viability **(B)**, and cell death **(C)** detected by SYTOX Green fluorescence dye in *C. reinhardtii* cells under an ML (750 μmol m^–2^ s^–1^) condition in the presence or absence of 400 μM cPTIO for 24 h. The data are expressed as the mean ± SD (*n* = 3) from three independent biological replicates and the vertical bar on the symbols represents SD. Different letters indicate the statistical significance set at *P* < 0.05 according to ANOVA.

Then, NO donors were administered under the ML condition to clarify whether NO could increase the susceptibility of algal cells under the ML condition. The results depended on the concentration of NO donors and the treatment time. Both cell density (Figure 11A) and viability (Figure 11B) were not affected by the presence of 0.05 mM GSNO or 0.05 mM SNAP but showed a decrease in response to 0.1 mM NO donor treatment. The cell death detected using SYTOX-Green fluorescence did not appear under treatment with 0.05 mM NO donors whereas it exhibited a marked increase under treatment with 0.1 mM NO donors (Figure 11C). The presence of cPTIO can suppress the negative effect of 0.1 mM NO donor treatment on cell activity (Figure 11C). The present findings demonstrate that supplementation with NO confers susceptibility of *C. reinhardtii* cells to moderate HL illumination.

**FIGURE 12:**
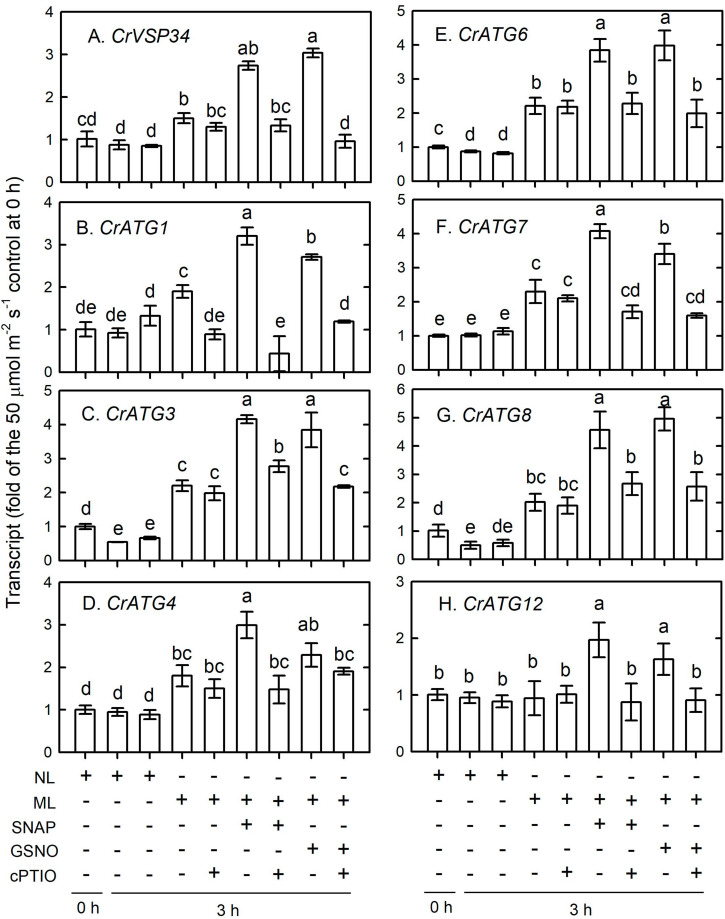
The time-course changes in CrVPS34 and CrATG transcripts abundance in *Chlamydomonas reinhardtii* cells in response to 0.1 mM SNAP or 0.1 mM GSNO treatment under an ML (750 μmol m^–2^ s^–1^) condition for 3 h. **(A)** CrVPS34. **(B)** CrATG1. **(C)** CrATG3. **(D)** CrATG4. **(E)** CrATG6. **(F)** CrATG7. **(G)** CrATG8. **(H)** CrATG12. The data are expressed as the mean ± SD (*n* = 3) from three independent biological replicates and different letters indicate the statistical significance set at *P* < 0.05 according to ANOVA.

The transcripts abundance of CrVPS34 (Figure 12A), CrATG1 (Figure 12B), CrATG3 (Figure 12C), CrATG4 (Figure 12C), CrATG6 (Figure 12E), CrATG7 (Figure 12F), and CrATG8 (Figure 12G) slightly increased 3 h after ML treatment and their increase can be enhanced by the supplementation with 0.1 mM NO donors, and this enhancement can be suppressed in the presence of cPTIO. Among the CrATG genes, the transcript abundance of CrATG12 was not increased by exposure to ML treatment, while the application of 0.1 mM NO donors induced an increase of its expression and this increase was also suppressed in the presence of cPTIO (Figure 12H).

### Interaction of NO and H_2_O_2_ for Autophagy Induction and Cell Death

**FIGURE 13:**
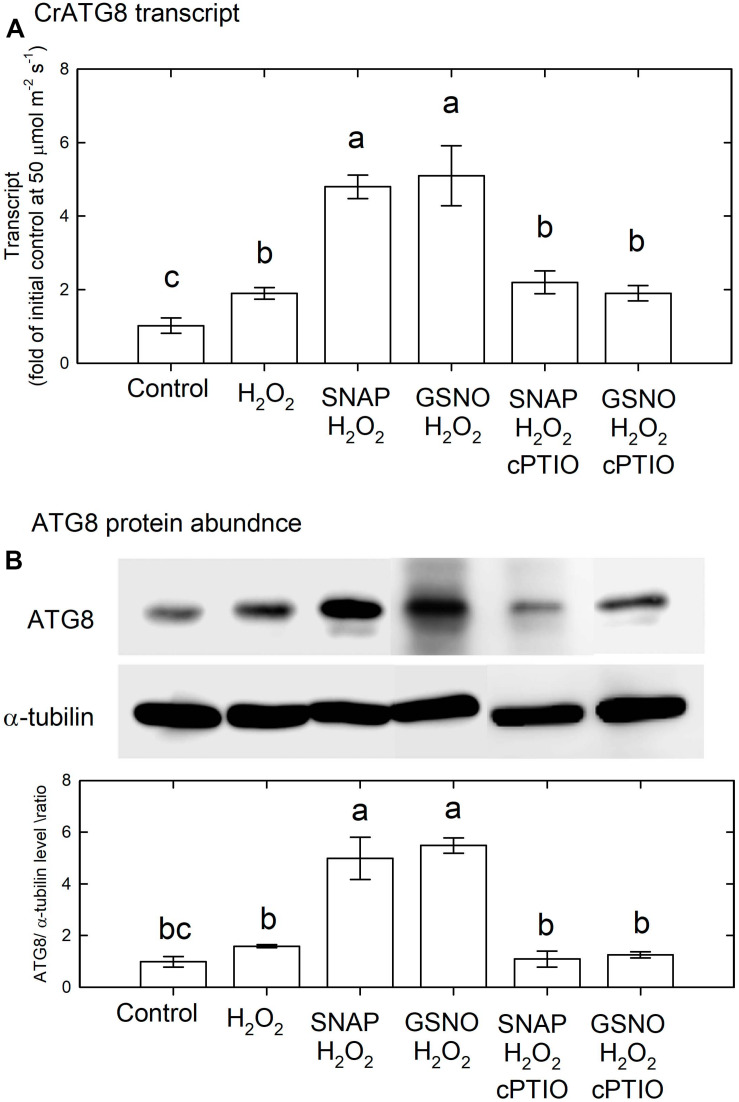
The effect of SNAP (0.1 mM), GSNO (0.1 mM), and 1 mM H_2_O_2_ treatment on the CrATG8 transcript abundance **(A)** and ATG8 protein abundance **(B)** of *Chlamydomonas reinhardtii* cells under an NL condition (50 μmol m^–2^ s^–1^) for 3 h. The cPTIO (0.4 mM) was added to the SNAP + H_2_O_2_ and GSNO + H_2_O_2_ treatments. The data are expressed as the mean ± SD (*n* = 3) from three independent biological replicates and different letters indicate the statistical significance set at *P* < 0.05 according to ANOVA.

The above results indicate that NO may interact with H_2_O_2_ for the induction of autophagy and cell death in *C. reinhardtii* cells. Therefore, NO donors at a concentration of 0.1 mM were applied together with 1 mM H_2_O_2_ under an NL condition for 3 h. The results shown in Figure 13A indicate that 1 mM H_2_O_2_ slightly increased the transcript abundance of CrATG8, while the combination of H_2_O_2_ and NO donors induced a marked increase in CrATG8 transcript abundance, which was inhibited in the presence of cPTIO. The ATG8 protein abundance in response to H_2_O_2_ treatment for 3 h slightly increased but the combination of H_2_O_2_ and NO donors induced a significant increase (Figure 13B).

**FIGURE 14:**
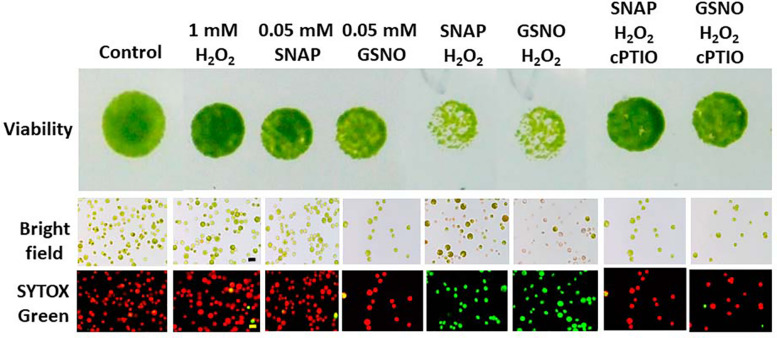
The effect of SNAP (0.1 mM), GSNO (0.1 mM), and 1 mM H_2_O_2_ treatment on the growth (cell density) **(A)** and viability **(B)** of *Chlamydomonas reinhardtii* cells under an NL condition (50 μmol m^–2^ s^–1^) for 24 h. The cPTIO (400 μM) was applied to the SNAP + H_2_O_2_ and GSNO + H_2_O_2_ treatments. The data are expressed as the mean ± SD (*n* = 3) from three independent biological replicates and different letters indicate the statistical significance set at *P* < 0.05 according to ANOVA.

The cell viability and cell death were also determined in response to H_2_O_2_ and/or NO donors in the presence or the absence of cPTIO under an NL condition for 24 h. We found that 1 mM H_2_O_2_ treatment did not affect cell viability and cell death, while 0.05 mM NO donors also did not affect cell viability and cell death (Figure 14). However, the combination of H_2_O_2_ and NO donor treatment induced a significant decrease in cell viability and resulted in significant cell death, which could be inhibited in the presence of 0.4 mM cPTIO (Figure 14). This demonstrated that NO interacts with H_2_O_2_ in the induction of cell damage under an NL condition.

## Discussion

Treatment of *C. reinhardtii* cells with high intensity illumination of 1,600 μmol m^–2^ s^–1^ triggered an NO burst during 3–5 h. An exposure to extremely high-light illumination at 3,000 μmol m^–2^ s^–1^ can also induce a significant NO production in *C. reinhardtii* cells (Chang et al., 2013). However, moderate HL illumination at 750 μmol m^–2^ s^–1^ intensity did not trigger NO production. This demonstrates that the generation of NO occurs only when the light intensity is higher than a certain threshold.

Recently, it has been shown that *C. reinhardtii* can metabolize NO to N_2_O and this metabolism is dependent on photosynthetic electron transport. These findings are supported by the data obtained using treatment with 3,4-dichlorophenyl-1,1-dimethylurea (DCMU), a photosystem II (PSII) inhibitor, or 2,5-dibromo-3-methyl-6-isopopyl-p-benzoquinone (DBMIB), an inhibitor of electron flow between PSII and PSI (Burlacot et al., 2020). This raises the possibility that the conversion of NO to N_2_O may lead to the disappearance of NO under the 750 μmol m^–2^ s^–1^ condition. However, we found that the application of 10 M DCMO or 2 M DBMIB did not increase NO production under the 750 μmol m^–2^ s^–1^ condition (data not shown). Thus, undetectable NO under the 750 μmol m^–2^ s^–1^ condition is not due to the degradation of NO to N_2_O.

The present results that the presence of L-NAME or tungstate cannot suppress the induction of NO production in *C. reinhardtii* cells by HL illumination suggests that routes other than the NOS and NR pathways are responsible for the HL-induced NO synthesis in *C. reinhardtii* cells. So far, whether NOS exists in plants is under debate, and the presence of NOS has not been verified in *C. reinhardtii*, as we know. Furthermore, the strain CC125 used in this study is the 137C *mt*− carrying the nit1 and nit2 mutations (*mt*− *nit1 nit2*) that lacks NR and does not express the proteins required for nitrate assimilation (NIA1, NRT2.1, NRT2.2, NII1, NAR2, and NAR1.1) (Galván and Fernández, 2001). The strain CC124, with genotype *mt*+ *nit1 nit2*, also exhibited a similar NO production pattern under 1,600 μmol m^–2^ s^–1^ illumination (Supplementary Figure S2). This suggests that the HL-induced NO production in *C. reinhardtii* cells is independent of NR. In fact, several studies have shown NO production in NR-defective *C. reinhardtii* mutants under hypoxia conditions (Hemschemeier et al., 2013) or in *C. reinhardtii* wild-type strain 137C (+) treated with MP (Yordanova et al., 2010). However, Wei et al. (2014) reported that the NR-defective *C. reinahrdtii* mutants produce less NO in response to nitrogen starvation, while a significant NO release is found in strains of the genotype NIT1 NIT2 with normal NR activity. It is therefore suggested that the NR-dependent route is responsible for the production of NO from *C. reinahrdtii* cells under nitrogen starvation (Wei et al., 2014). The above results indicate that the routes used for the synthesis of NO in *C. reinhardtii* cells are different not only between the strains with various genetic backgrounds but also between different treatment conditions. Our present findings demonstrate that *C. reinahrdtii* lacking NR can generate marked NO production via NR- and NOS-independent routes under illumination at a very high intensity of 1,600 μmol m^–2^ s^–1^.

The evidence suggests that NO contributes to the activation of autophagy in *C. reinhardtii* cells by HL illumination at 1,600 μmol m^–2^ s^–1^ intensity. When the marked NO burst occurs during 3–5 h following HL illumination, *C. reinhardtii* cells concomitantly exhibited marked autophagy induction, supported by an increase in the abundance of CrVPS34 and CrATG transcripts and CrATG8 protein, a *Chlamydomonas* autophagy marker (Pérez-Pérez et al., 2010). The results of Pérez-Pérez et al. (2012a) also showed that there is a transient activation of autophagy with a peak at 6 h in *C. reinhardtii* cells subjected to 1,200 μmol m^–2^ s^–1^ illumination, reflected by increased CrATG8 protein abundance detected using western blot and immunofluorescence analyses. More importantly, the present finding that the induction of autophagy by illumination at a higher light intensity up to 1,600 μmol m^–2^ s^–1^ can be suppressed in the presence of a NO scavenger, cPTIO, which demonstrates that the activation of autophagy under the 1,600 μmol m^–2^ s^–1^ condition can be attributable to NO. Similarly, the VHL (3,000 μmol m^–2^ s^–1^)-induced increase in NO production (Chang et al., 2013) and the abundance of CrATG8 protein (Supplementary Figure S4) and the CrVPS34, CrATG, and CrTOR transcripts (Supplementary Figure S5) in *C. reinhardtii* can be suppressed by cPTIO treatment.

Furthermore, treatment with GSNO or SNAP at either 0.05 or 0.1 mM under the 50 μmol m^–2^ s^–1^ condition is able to trigger autophagy, reflected by the increase of CrVPS34 and CrATG transcripts abundance and CrATG8 protein abundance, which is suppressed in the presence of cPTIO. Obviously, NO can trigger autophagy without other factors derived from HL illumination. As far as we know, this is the first time that NO has been shown to be involved in the modulation of HL-induced autophagy in *C. reinhardtii* cells through upregulation of CrVPS34 and CrATG gene expression. The effect of NO on the induction of autophagy is well-known in animal systems (Barsoum et al., 2006; Yang et al., 2008; Sarkar et al., 2011; Duana et al., 2013; Tripathi et al., 2013).

The upregulation of CrVPS34 and CrATG expression by NO and the suppression of their expression by cPTIO indicated that the signaling components are involved in the NO-mediated activation of autophagy under HL illumination. Among the core autophagy machineries, autophagy induction is driven by the association of ATG1 kinase with dephosphorylated ATG13 and other regulatory ATG proteins for the construction of a preautophagosomal structure (Kamada et al., 2000). Subsequently, vesicle nucleation is initiated by the activation of vacuolar-protein-sorting (VPS) 34 PI3K complex containing ATG6 and other VPS proteins (Thompson and Vierstra, 2005). VPS34 is the Class III PI3K, which is conserved from yeasts and plants to mammals (Engelman et al., 2006) for the phosphorylation of phosphatidylinositol to generate phosphatidylinositol 3-phosphate that is a phospholipid central to membrane trafficking processes, is required for the formation of the autophagosome (Itakura et al., 2008; Jaber et al., 2012). Here, the increase of CrVPS34, CrATG1, and CrATG6 transcripts abundance 3 h after HL illumination may indicate that the vesicle nucleation of the phagophore assembly site to form the autophagosome is executed in *C. reinhardtii* cells under HL stress. Subsequently, an increase in CrATG8 protein abundance and transcripts abundance of CrATG3, CrATG4, CrATG7, and CrATG8 function in the expansion of the autophagosomal membrane and its fusion with the lysosome/vacuolar membrane through the ubiquitin-like protein conjugation system (Ohsumi, 2001; Thompson and Vierstra, 2005; Hanada et al., 2007; Nakatogawa et al., 2007; Xie et al., 2008), which implies the implementation of a mature autophagosome and autolysosome formation in *C. reinhardtii* cells under the control of NO generated under HL illumination.

Our findings indicate that the expression of CrATG8 is regulated by NO and other factors under very HL conditions of 1,600 μmol m^–2^ s^–1^. An increase in CrATG8 transcript abundance by prolonged HL illumination (5 h) is partially inhibited in the presence of cPTIO. Even when cPTIO was administered at a concentration up to 800 M, the CrATG8 transcript abundance still remained at a level approximately 24% of the HL treatment without cPTIO application (data not shown). This may reflect that other factors work with NO for the induction of autophagy in *C. reinhardtii* under the 1,600 μmol m^–2^ s^–1^ condition. Pérez-Pérez et al. (2012a) have shown increased levels of ROS in the chloroplast and a marked increase in autophagic activity due to the absence of photoprotection in *C. reinhardtii* cells. This may suggest that ROS are also the factors that induce CrATG8 expression (protein abundance) in *C. reinhardtii* cells exposed to the 1,600 μmol m^–2^ s^–1^ condition. In addition, their study showed that the treatment with 1 mM H_2_O_2_ under a 20–30 μmol m^–2^ s^–1^ condition or a dark condition, or 1 M MV under a 20–30 μmol m^–2^ s^–1^ condition for 8 h, can increase the CrATG8 protein abundance (Pérez-Pérez et al., 2012a). In addition, in a study of the responses of a starchless (*sta6*) mutant to oxidative stress and the induction of autophagy in *C. reinhardtii* cells, Tran et al. (2019) showed that there is a dose-dependent expression pattern of ATG8 protein detected using western blot analysis with a linear increase in ATG8 protein level in response to a gradual increase in H_2_O_2_ concentration from 0, 0.25, 0.50, to 1 mM. Here, we found that the H_2_O_2_ concentration was significantly increased by exposure to 1,600 μmol m^–2^ s^–1^ illumination (Supplementary Figure S6). Moreover, we also observed that the exposure of *C. reinhardtii* cells to 1 mM H_2_O_2_ for 3 h under an NL condition increased the ATG8 protein abundance and CrATG8 transcript abundance without any adverse effect on cell viability. The application of NO together with H_2_O_2_ induces significant increases in ATG8 protein and CrATG8 transcript abundances, which can be suppressed by cPTIO treatment. In addition, although autophagy is not occurring under the ML condition, the administration of 0.1 mM NO donors can induce autophagy under the ML condition. This provides evidence that, in addition to NO, H_2_O_2_ is also involved as a signal for the activation of autophagy by 1,600 μmol m^–2^ s^–1^ illumination of *C. reinhardtii* cells.

In addition to the induction of autophagy, 1,600 μmol m^–2^ s^–1^ illumination resulted in cell death (bleaching) after 5 h. The prevention of HL-induced cell death by cPTIO treatment supports the hypothesis that NO is involved in the HL-induced cell death. In addition, our evidence shows that other factors, such as ROS, may interact with NO for the induction of cell death in *C. reinhardtii* cells under lethal HL stress (1,600 μmol m^–2^ s^–1^). H_2_O_2_ has been shown to be involved in plant cell death (Houot et al., 2001). However, the effect of H_2_O_2_ on the induction of cell death works in a concentration-dependent manner. Murik et al. (2014) provided evidence that the growth rate of *C. reinhardtii* cells is not affected by H_2_O_2_ at concentrations below 2 mM whereas it was markedly retarded on exposure to higher H_2_O_2_ concentrations in a range between 5 and 10 mM. Here, illumination of *C. reinhardtii* cells at an intensity of 1,600 μmol m^–2^ s^–1^ can induce significant H_2_O_2_ accumulation (Supplementary Figure S6). The evidence shows that NO bursts along with H_2_O_2_ accumulation occur as the 1,600 μmol m^–2^ s^–1^ illumination advances to 5 h (Figure 1A), and treatment with 0.1 mM NO donors can induce a transient growth depression and cell death under an NL condition. More importantly, the presence of the NO scavenger, cPTIO, can effectively prevent growth retardation and cell death caused by the 0.1 mM NO donors. Furthermore, under the ML condition (750 μmol m^–2^ s^–1^), the application of 0.1 mM NO donors can cause cell death and growth retardation, which is prevented by cPTIO treatment. Because H_2_O_2_ also accumulates under the ML condition (750 μmol m^–2^ s^–1^), this again supports that the NO released from the NO donors interacts with H_2_O_2_ for the induction of cell death.

To further prove this proposal, we treated *C. reinhardtii* cells with 1 mM H_2_O_2_ and 0.05 mM NO donors together under an NL condition to check whether the application of both chemicals can cause cell death without HL interference. Our results confirmed that exogenously applied NO acts synchronously with accumulated H_2_O_2_ to enhance the sensitivity of *C. reinhardtii* cells to moderate HL illumination, leading to a decrease in cell viability and partial cell death, whereas the treatment with 1 mM H_2_O_2_ alone could not affect cell growth and the treatment with 0.05 mM NO donors also did not impact cell activity. Overall, these lines of evidence demonstrate that excessive NO can interact with H_2_O_2_ for the induction of cell death in *C. reinhardtii* cells under lethal HL illumination. In higher plants, NO bursts after a pathogen attack initiate gene-to-gene defense responses and they also interact with H_2_O_2_ and other signals in triggering PCD (Delledonne et al., 2001; Hong et al., 2008; Floryszak-Wieczorek and Arasimowicz-Jelonek, 2010). In the investigation of the MP-induced cell death in *C. reinhardtii* cells, Yordanova et al. (2010) have identified that NO cooperates with ethylene for the induction of cell death caused by MP. Obviously, NO interacts with other factors for the induction of cell death in *C. reinhardtii* cells, depending on the different stress conditions.

It has long been known that NO exhibits a dual function, beneficial or harmful, in stress conditions depending on its concentration, the type of tissue, age or physiological status, its ability to interact with other signaling molecules, and the type of stress (Delledonne et al., 2001; Arasimowicza and Floryszak-Wieczorek, 2007; Das et al., 2012). For instance, NO at a high concentration causes membrane breakdown, DNA fragmentation, and cell death (Pedroso et al., 2000; Yamasaki, 2000; Romero-Puertas et al., 2004). NO is also involved in the regulation of hypersensitive cell death (Clarke et al., 2000; de Pinto et al., 2002) and stress-induced cell death (Ahlfors et al., 2009; de Michele et al., 2009) in higher plants. In the diatoms, treatment with NO donors mimics aldehyde-induced cell death (Vardi et al., 2006). A negative role of NO on algal growth has been shown in the unicellular green alga *Micrasterias denticulate* (Brébisson) by exogenous application of a NO donor. The NO suppresses algal cell growth via the inhibition of the activity of enzymes involved in the secretary pathway (Lehner et al., 2009). NO also triggers cell death in algae; for example, aldehyde-induced cell death in diatoms (Vardi et al., 2006) and the heat-induced cell death of symbiotic alga *Symbiodinium microadriaticum* Freudenthal (Bouchard and Yamasaki, 2008). Previously, we have reported that mass generation of NO at a higher concentration under a 3,000 μmol m^–2^ s^–1^ condition contributes to the induction of cell death of *C. reinhardtii* cells (Chang et al., 2013). Therefore, NO overproduced in *C. reinhardtii* under extreme HL illumination opens the way to cell death. In contrast, our current study shows that, at lower concentrations, NO does not affect cell growth or cause permanent cell damage to *C. reinhardtii* cells. Treatment with 0.05 mM NO donors does not affect cell activity while 0.1 mM NO donors can trigger a transient effect on cell activity, followed by a rapid recovery after prolonged culture.

In addition to the concentration effect, the promotion or prevention of cell death is also dependent on the plant systems and conditions. In some plant tissues, NO counteracts PCD. For example, NO delays the onset of GA-induced PCD in barley (*Hordeum vulgare* cv Himalaya) aleurone layers (Beligni et al., 2002). In a study of a carotenoid deficient mutant, *lts1-204*, in the regulation of autophagy induction in *C. reinhardtii* cells by oxidative stress, Pérez-Pérez et al. (2010) demonstrated that 1,200 μmol m^–2^ s^–1^ illumination transiently activated autophagy in wild-type *Chlamydomonas* cells as part of an adaptation response to oxidative stress. In contrast, our current investigation implies that excessive NO generated under lethal HL illumination at the intensity of 1,600 μmol m^–2^ s^–1^ cooperates with highly accumulated H_2_O_2_ for the modulation of the signaling pathways, causing activation of autophagy, which leads to the death of *C. reinhardtii* cells.

First, we showed that autophagy induction is positively correlated with cell death in response to a lethal HL condition. This is in contrast to other conditions, such as nutrient starvation, where autophagy is considered to act as a protective process for *C. reinhardtii* against stress conditions. The review article of Pérez-Pérez et al. (2012b) has concluded from many studies that autophagy is conserved in plants and algae and plays a role in the adaptation to different stress conditions for the recycling of ROS-mediated oxidatively damaged macromolecules and organelles. Similarly, in hypersensitive plants, autophagy functions in the prevention of PCD (Greenberg, 2005). In contrast, in a study of the pathogen causing rice blast disease (*Magnaporthe grisea*), the use of an *Mgatg8* knockout line confirmed that autophagy is responsible for death of the spores (Veneault-Fourrey et al., 2006). Furthermore, in animal cells, certain forms of cell death can be inhibited by treatment with autophagic inhibitors or by reduced ATG gene expression (Shimizu et al., 2004; Yu et al., 2004). It has also been shown that autophagy-induced cell death is linked to unbalanced ROS production in animal cells. Using the mammalian L929 cell line, a widely studied model for TNF-α-induced cell death, caspase inhibitor, benzyloxycarbonyl-valyl-alanyl-aspartic-acid (*O*-methyl)-fluoromethylketone (zVAD) treatment results in selective autophagic degradation of catalase, a key enzymatic ROS scavenger, and in turn, this leads to marked ROS accumulation and ultimately causes non-apoptotic death (Yu et al., 2006). Thus, these findings imply that autophagy initiates and participates directly in the death process in animal systems. In the green alga *Chlamydomonas*, our current evidence shows that NO and H_2_O_2_ interact in the induction of autophagic cell death when exposed to lethal HL conditions. In the near future, a genetic investigation using ATG mutants is necessary to identify the network linking autophagy and cell death in *Chlamydomonas* under lethal HL stress.

In conclusion, the present study is the first to clearly demonstrate the interaction between NO and H_2_O_2_ in the activation of autophagic cell death in *C. reinhardtii* under lethal HL stress. This finding provides a background for further examination of the role of NO as a signaling molecule in the control of autophagy induction and the role of ROS interactions with NO during the induction of cell death in *C. reinhardtii* under lethal HL stress.

## Data Availability Statement

The raw data supporting the conclusions of this article will be made available by the authors, without undue reservation, to any qualified researcher.

## Author Contributions

H-LC and EK performed the physiological analysis, RNA extraction, cDNA preparation, and qPCR. S-TL contributed to the preparation of reagents and the determination of biochemical and physiological parameters. T-ML conceived and designed the experiments, interpreted the data, and wrote the manuscript. All authors discussed and reviewed this manuscript.

## Conflict of Interest

The authors declare that the research was conducted in the absence of any commercial or financial relationships that could be construed as a potential conflict of interest.

## Footnotes

1 http://chlamycollection.org/
