# Supplementary material for: High Light-Induced Nitric Oxide Production Induces Autophagy and Cell Death in Chlamydomonas reinhardtii
Source: Front Plant Sci. 2020 Jun 10;11:772. doi: 10.3389/fpls.2020.00772 (PMC7298128; doi:10.3389/fpls.2020.00772)
Supplement: Supplementary file 1 [file Data_Sheet_1.PDF]

SUPPLEMENTARY TABLE

Supplementary Table S1. Primers for the *CrATG1, CrATG3, CrATG4, CrATG6, CrATG7, CrATG8, CrATG12, CrVPS34,* and internal control genes (*CrUBC, CrEF-1 α*) used for real-time PCR analysis.

Gene	Forward primer sequence (5' → 3')	Reverse primer sequence (5' → 3')
CrUBC	CATTAGAGGCGGGCAAA	TATCGTCATCGTGGTTGTGTAT
CrEF-1α	TGGTACAGGGTCGCTTG	TGCTTCAGCGACACGAG
CrATG1	CGGAAGAGGACTAAAGAGACACT	GTGCAGAGCCGACAACC
CrATG3	GCGTGTGAGATCCTTTCC	GATAGGCTTTCATGTGCTGC
CrATG4	GGATATGCAGAGAGTGCTT	CTCACAACCGACAATACCAAA
CrATG6	CATGCCGTTCAACTTCC	GCGTCCTTGTTTAGTGTCAG
CrATG7	GTGTCAGCACGATAGGTAG	CTCAAGCTGCTTACCATACT
CrATG8	ACTAATGTGGGCTCAGTTT	CCTTCCCAGCGCATTAC
CrATG12	TGACTCTGCGGTTGGAA	TAGGGCACATTGCAGGAT
CrVPS34	AGCTGGATATTCGCTCTG	ATCCAAGTAGCTCGTATCG

SUPPLEMENTARY FIGURE

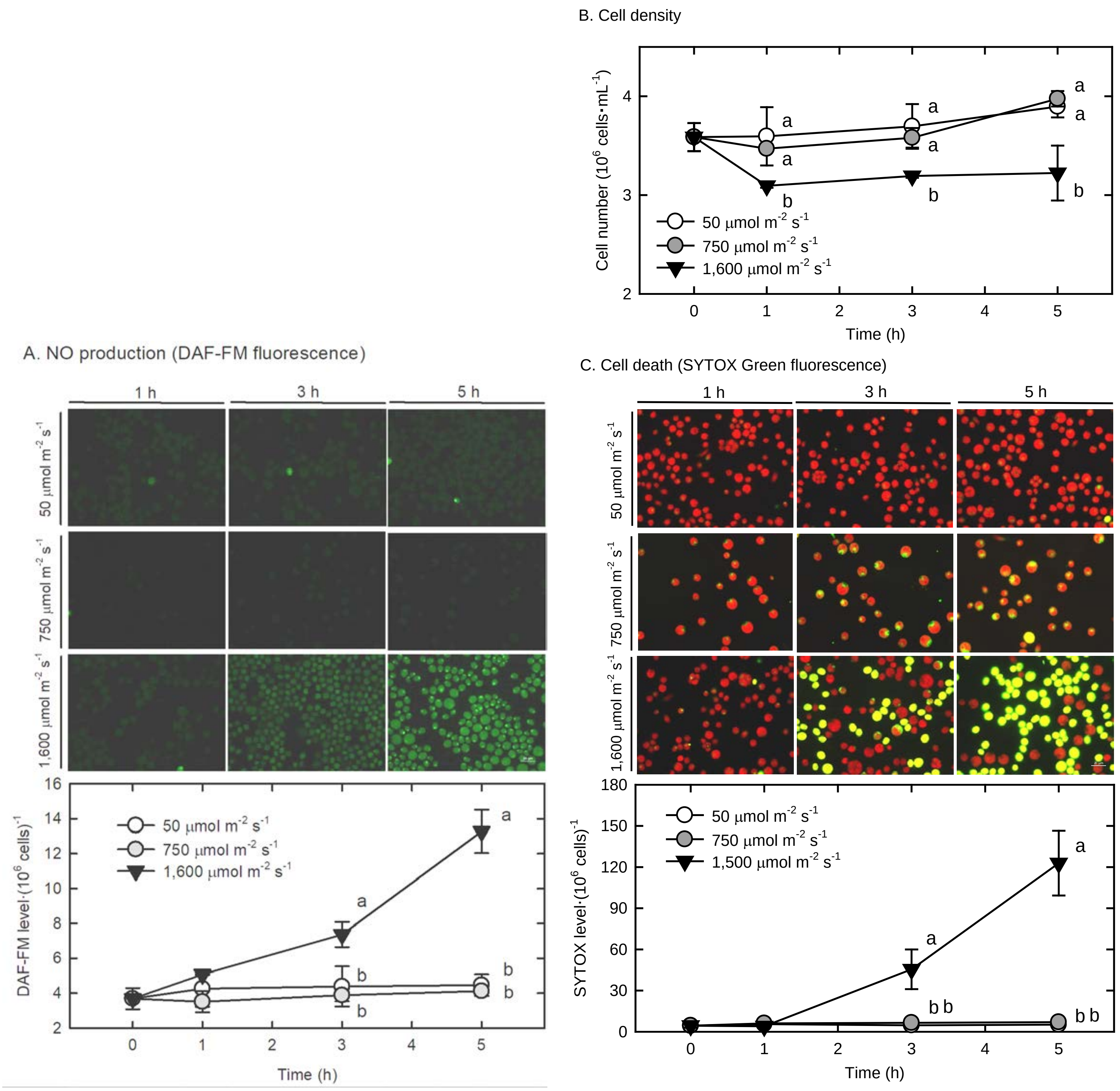

Supplementary Figure S1. The NO production (A), cell growth (B), and cell death (C) in *Chlamydomonas reinhardtii* cells illuminated at 50, 750 or 1,600 μmol·m^{-2}·s^{-1}. (A) Microscopic observation of DAF-FM fluorescence (upper part) and quantitation of relative changes in NO production (lower part). (B). Cell density. (C). Microscopic observation of SYTOX Green fluorescence (upper part) and their relative quantitation (lower part). The data are expressed as the mean ± SD (n=3) and the vertical bar on each symbol represents SD. Different letter indicates the statistical significance set at $P < 0.05$ according to ANOVA analysis.

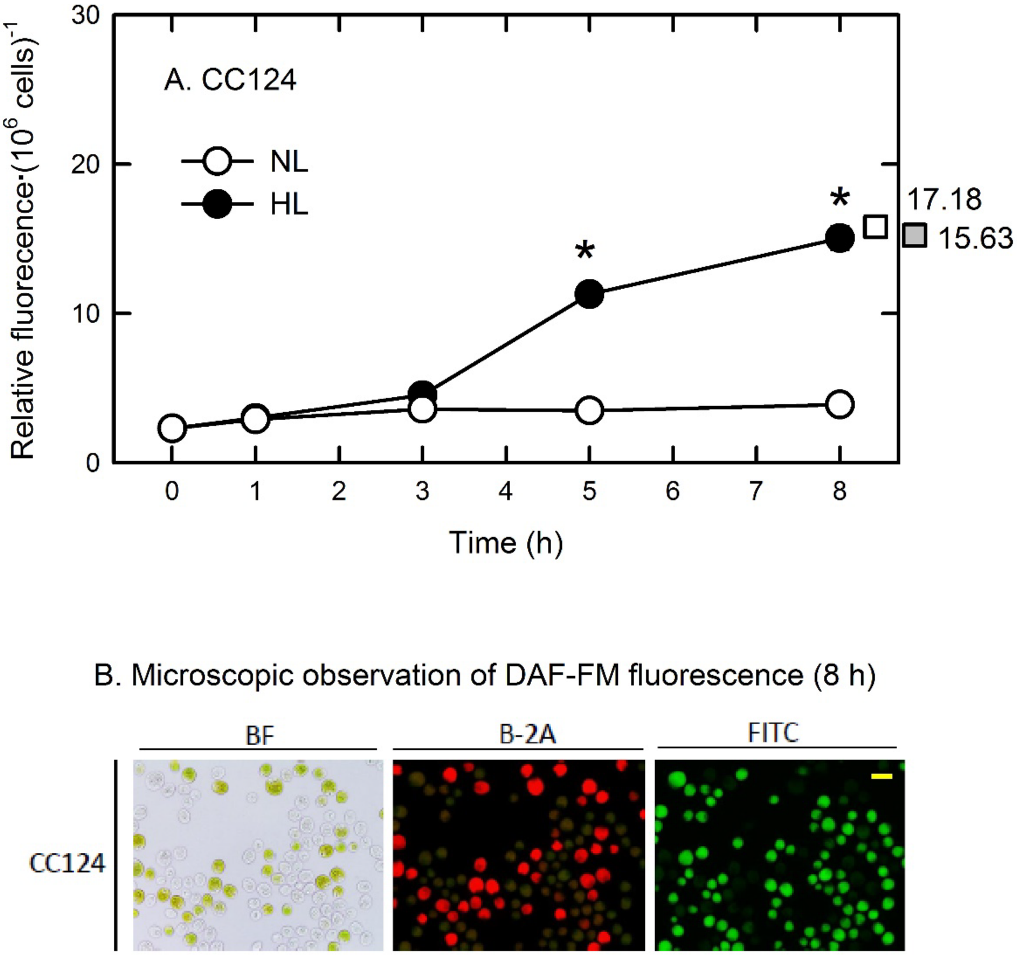

Supplementary Figure S2. DAF-FM acetate detection of NO production in the cells of *Chlamydomonas reinhardtii* strain CC124 under NL (50 μmol·m^{-2}·s^{-1}) and HL (1,600 μmol·m^{-2}·s^{-1}) conditions. (A) Quantitation of NO production. (B) Microscopic observation of DAF-FM fluorescence after 5 h of treatment. The data in (A) are expressed as the mean ± SD (n=3) and the vertical bar on each symbol represents SD. * indicates significant differences between NL and HL treatment (*t*-test, $P < 0.05$). In (B), BF represents bright field, B-2A represents the autofluorescence of cells, and FITC represents DAF-FM fluorescence. Yellow bar indicates 20 μm.

The mean of numbers beside the squares in Supplementary Figure S2A mean:

1. Open square (□), the relative fluorescence of DAF-FM of 500 μM N^{ω}-nitro-L-arginine methyl ester (L-NAME) treatment after 8 h.
2. Solid square (■), the relative fluorescence of DAF-FM of 300 μM tungstate treatment after 8 h.

The treatment with L-NAME (*t*-test, P=0.9305) and tungstate (*t*-test, P=0.8538) did not show significant difference to the 8-h HL treatment in the absence of chemicals.

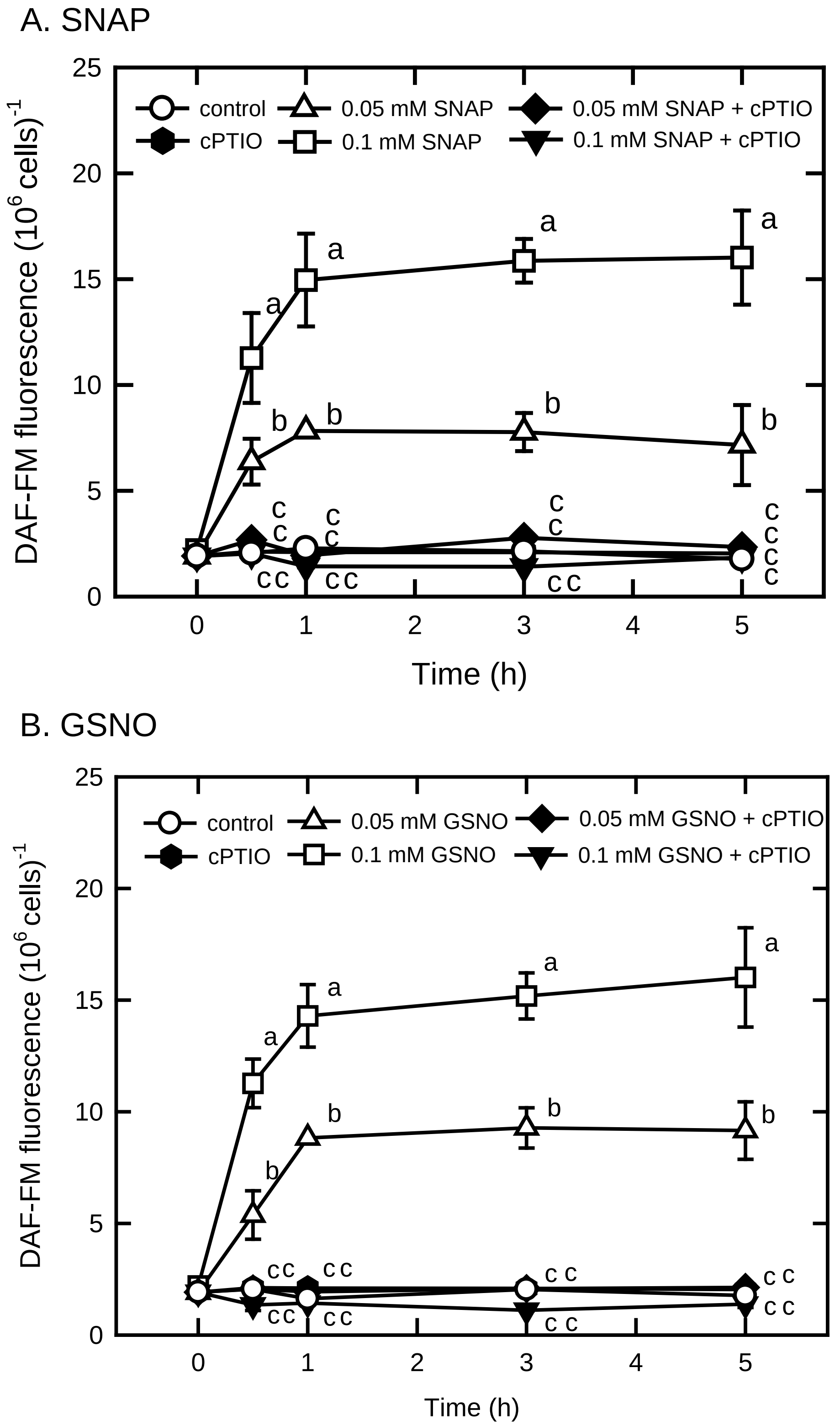

Supplementary Figure S3. Release of NO from SNAP and GSNO and the effect of 400 µM cPTIO on scavenging NO generated from SNAP- or GSNO-treated *Chlamydomonas reinhardtii* cells under NL condition (50 µmol·m^{-2}·s^{-1}). (A) SNAP treatment in the presence or absence of cPTIO. (B) GSNO treatment in the presence or absence of cPTIO. The data are expressed as the mean ± SD (n=3) and different letter indicates the statistical significance set at $P < 0.05$ according to ANOVA analysis ($P < 0.05$).

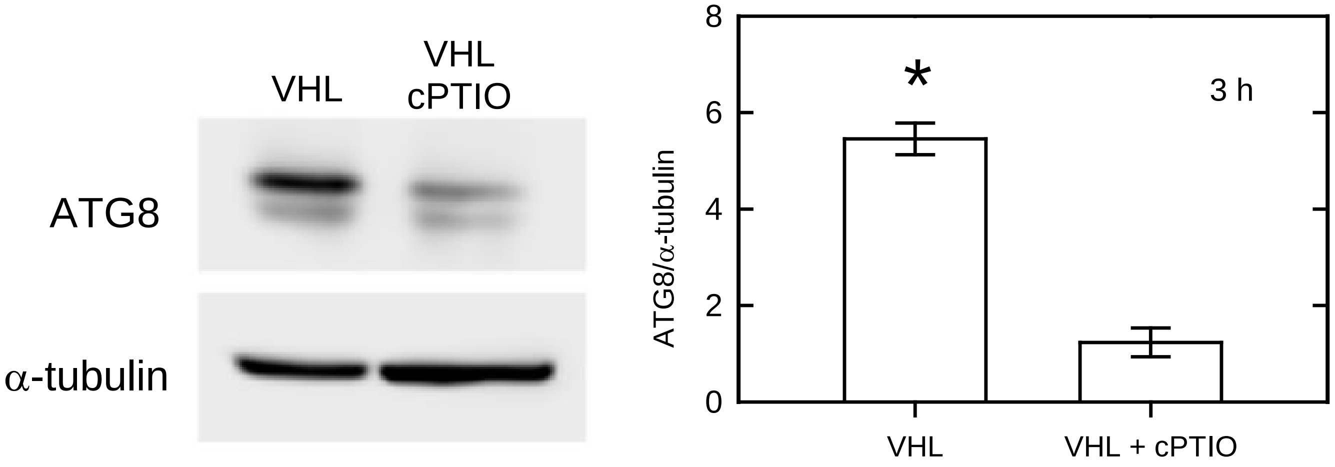

Supplementary Figure S4. Induction of CrATG8 protein by illumination at very high light (VHL) intensity of 3,000 µmol·m^{-2}·s^{-1} in the presence or the absence of 400 µM cPTIO for 3 h. The immunoblot detection of CrATG8 protein showed the inhibition of VHL-induced CrATG8 protein increase by 400 µM cPTIO. The data for relative expression of CrATG8 protein are expressed as the mean ± SD (n=3 for three biological replicates used for western blot assay) and * indicates significant differences between VHL and VHL+cPTIO treatments using the Student's *t*-test ($P < 0.05$).

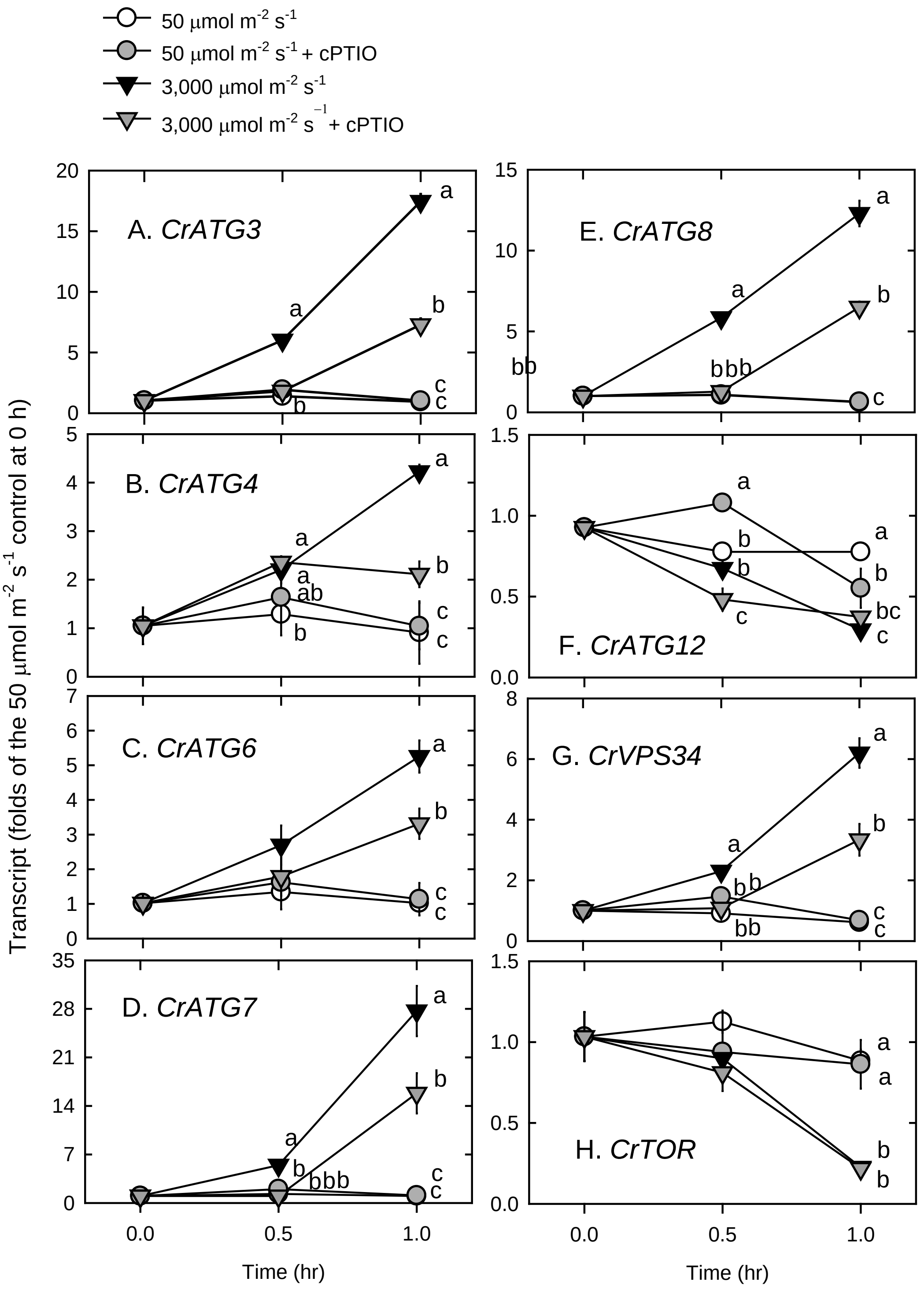

Supplementary Figure S5. CrVPS34, CrATG, and CrTOR transcript abundances of *Chlamydomonas reinhardtii* cells in response to 3,000 μmol·m^{-2}·s^{-1} illumination (VHL) in the presence or the absence of 400 μM cPTIO. (A) CrATG3. (B) CrATG4. (C) CrATG6. (D) CrATG7. (E) CrATG8. (F) CrATG12. (G) CrVPS34. (H) CrTOR. The data are expressed as mean ± SD (n=3) and the vertical bar on each symbol represents SD. different letter indicates the statistical significance set at $P < 0.05$ according to ANOVA analysis ($P < 0.05$). The reference gene is CrUBC (Supplementary Table S1). The forward primer of CrTOR is CGTGTTCTTGATTTGATGGTAG and the reverse primer is TGCATCCCTCAGGTGTC.

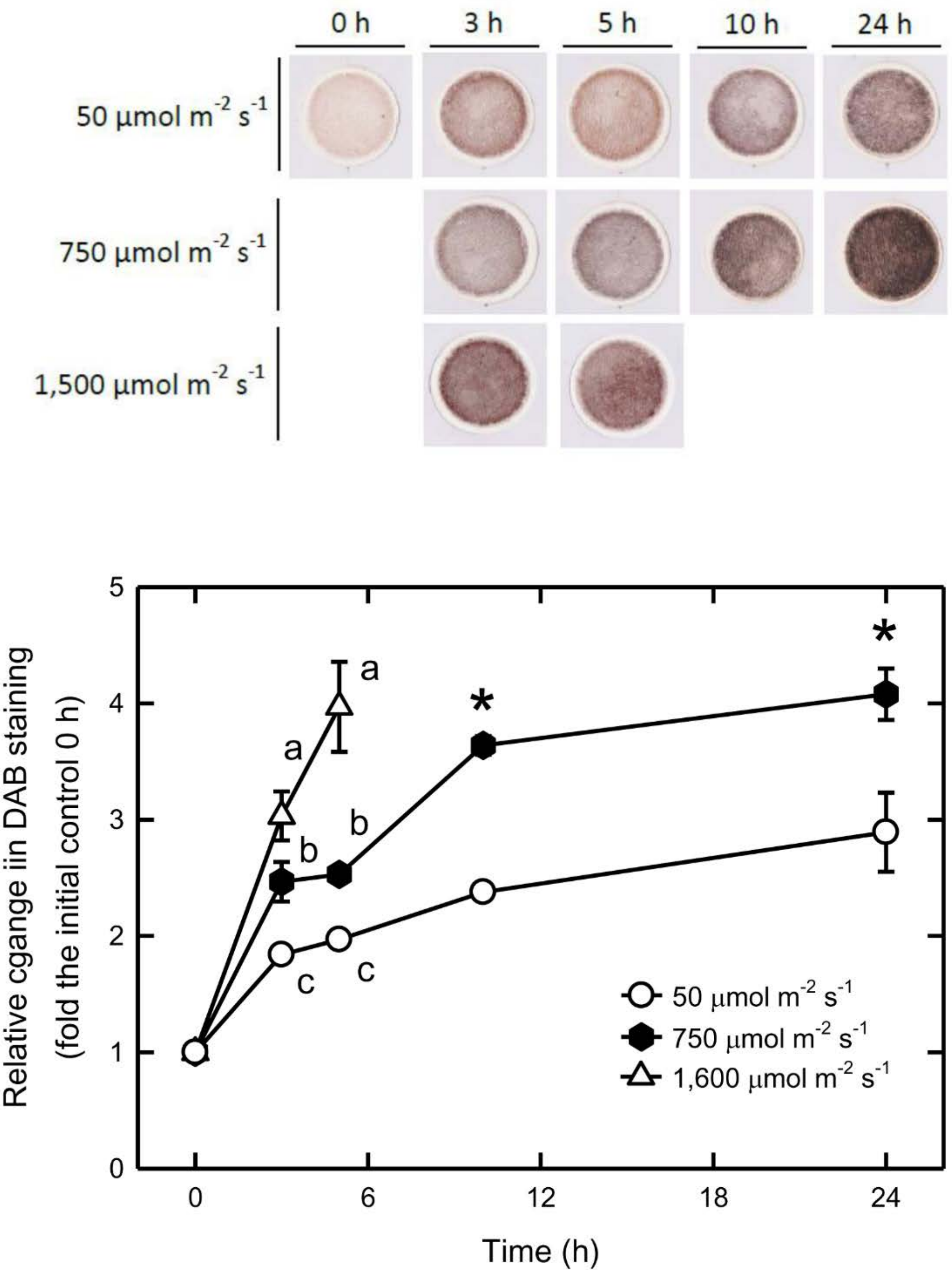

Supplementary Figure S6. Determination of H_2O_2 concentration by DAB staining of *Chlamydomonas reinhardtii* cells in response to 50 µmol m^{-2} s^{-1}, 750 µmol·m^{-2}·s^{-1}, or 1,600 µmol·m^{-2}·s^{-1}. The samples for 1,600 µmol·m^{-2}·s^{-1} treatment were assayed till 5 h because the algal cells bleached and died after 5 h (see Figure 1 in the main manuscript). The data are expressed as mean ± SD (n=3) and the vertical bar on each symbol represents SD. Different letters indicate significant difference among treatments during 0-5 h using Duncan's new multiple range test ($P < 0.05$), and * indicates significant difference between 50 and 750 µmol·m^{-2}·s^{-1} treatments during 10-24 h using Student's *t*-test ($P < 0.05$).

H_2O_2 was detected using 3,3-diaminobenzidine (DAB)-HCl (Sigma, St. Louis, MO, USA) according to the method of Hema et al. (2007) with some modifications. The cells were pretreated with DAB before high-intensity illumination. The cells were centrifuged at 3000 ×*g* (Centrifuge 5810R, Eppendorf AG, Hamburg, Germany) using a swing-bucket rotor (F-34-6-38, Eppendorf AG, Hamburg, Germany) for 3 min at room temperature. The pellet was re-suspended in new TAP medium containing 5 mM DAB for a 10-min incubation in the dark. Subsequently, the algal cells were illuminated under NL or HL condition. At each time point, the cells were filtered onto glass microfiber filters (diameter 45mm, GF/C, Whatman, GE Healthcare, Piscataway, NJ, USA). The pigments were completely removed following the wash of the filter discs twice with methanol. After drying, the filters were scanned as digital images and staining intensities were estimated using IMAGEJ software (free software from http://rsbweb.nih.gov/ij/index.html) and compared between treatments. To confirm that the browning of DAB dye by cellular accumulated H_2O_2,100 U mL^{-1} bovine liver catalase (Sigma-Aldrich, St. Louis, MO, USA) was added together with DAB dye in the culture medium. We found that catalase could effectively reduce the brown color of algal cells (data not shown). This indicates that the color development after DAB staining was mainly due to H_2O_2.

Hema, R., Senthil-Kumar, M., Shivakumar, S., Chandrasekhara R. P., and Udayakumar, M. (2007) *Chlamydomonas reinhardtii*: a model system for functional validation of abiotic stress responsive genes. *Planta* 226, 655–670. doi: 10.1007/s00425-007-0514-2

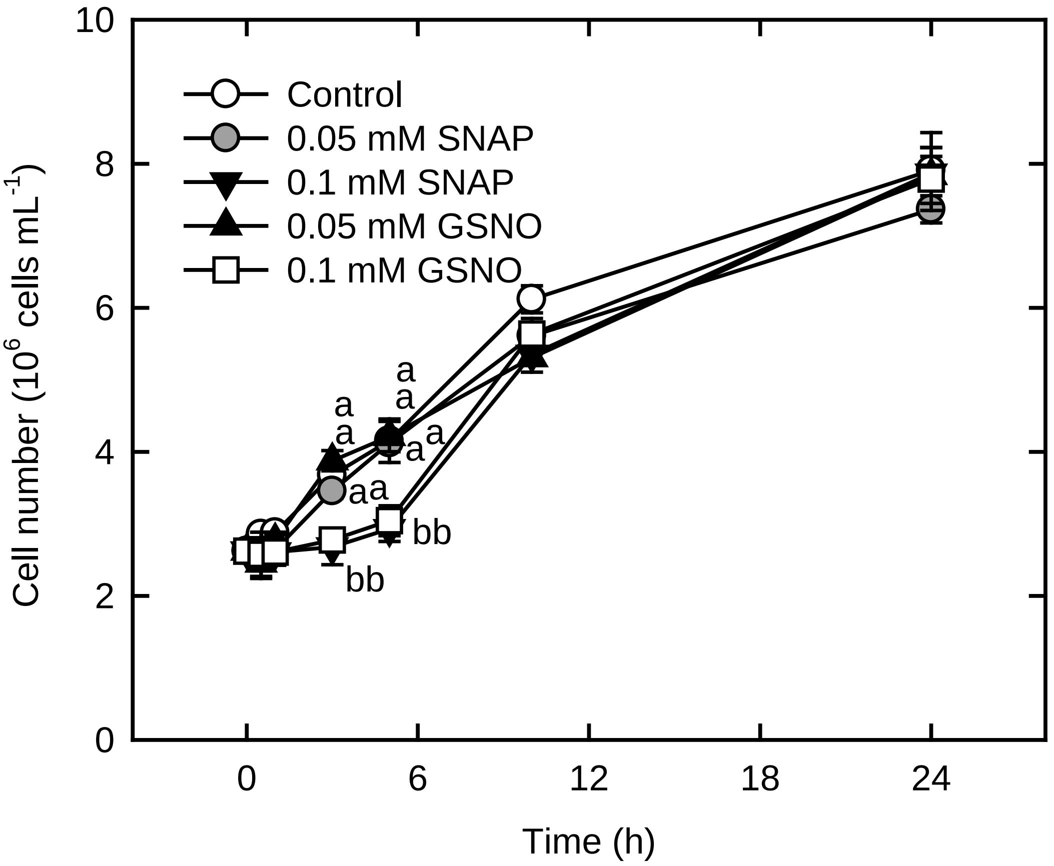

Supplementary Figure S7. The effects of NO donor, SNAP and GSNO on the changes in cell growth. The data are expressed as mean ± SD (n=3) and the vertical bar on each symbol represents SD. Different letters indicate significant difference among treatments using Duncan's new multiple range test ($P < 0.05$).
